# Chemistry, Biological Activities and In Silico Bioprospection of Sterols and Triterpenes from Mexican Columnar Cactaceae

**DOI:** 10.3390/molecules25071649

**Published:** 2020-04-03

**Authors:** Juan Rodrigo Salazar, Marco A. Loza-Mejía, Diego Soto-Cabrera

**Affiliations:** Design, Isolation, and Synthesis of Bioactive Molecules Research Group, Chemical Sciences Faculty, Universidad La Salle. Benjamín Franklin 45, Mexico City 06140, Mexico; diego.soto_cabrera.3180@student.lu.se

**Keywords:** Cactaceae, bioprospection, sterol, triterpene, bioactivity, in silico screening, inflammation, diabetes

## Abstract

The Cactaceae family is an important source of triterpenes and sterols. The wide uses of those plants include food, gathering, medicinal, and live fences. Several studies have led to the isolation and characterization of many bioactive compounds. This review is focused on the chemistry and biological properties of sterols and triterpenes isolated mainly from some species with columnar and arborescent growth forms of Mexican Cactaceae. Regarding the biological properties of those compounds, apart from a few cases, their molecular mechanisms displayed are not still fully understand. To contribute to the above, computational chemistry tools have given a boost to traditional methods used in natural products research, allowing a more comprehensive exploration of chemistry and biological activities of isolated compounds and extracts. From this information an in silico bioprospection was carried out. The results suggest that sterols and triterpenoids present in Cactaceae have interesting substitution patterns that allow them to interact with some bio targets related to inflammation, metabolic diseases, and neurodegenerative processes. Thus, they should be considered as attractive leads for the development of drugs for the management of chronic degenerative diseases.

## 1. Introduction

The word Cactaceae derived from the Greek, “*káctos*”, used in antiquity to name a species of thorny thistle, possibly the cardo *Cynara cardunculus* (Asteraceae), being used as a generic name by Carlos Linneo in 1753, for several plants of diverse sorts of the Cactaceae family [1]. This family, originally from America, groups about 1500 species. It is estimated that about 700 species grouped in 68 genera are present in Mexico, although there is considerable variability in these data [2,3,4].

The most apparent characteristic of cacti is the presence of the areola, considered as buds homologous to the axillary buds of the other dicotyledons. One main characteristic of buds or areolas is its capacity to form reduced leaves, flowers, new stems, thorns, glands, bristles, and hairs, even sometimes adventitious roots [2]. The ethnobotanical importance of cacti has been described extensively in the literature, including their use as food, medicine, among others. Some species of the family Cactaceae have been used as natural remedies for many centuries in Mexican traditional medicine. For example, peyote (*Lophophora williamsii*) has been used not only as a means of communication of man to the spiritual world but also as an analgesic and antirheumatic. On the other hand, *Ariocarpus kotschoubeyanus* has been used as an analgesic for bruises, the stems of *Pachycereus pecten-aboriginum* have been used to cure gastric ulcers, and as dressings to calm rheumatic pains and inflammatory processes, the latex of *Mammillaria heyderi* has been used by the Tarahumara’s community for earache and deafness. Parts of *Stenocereus thurberi* have been used to relieve pain in general [5].

Despite the widespread use of these species, in general, the literature concerning the chemistry, biological activities, or toxicity of these plant resources is scarce. This situation evidences the lack of studies, except for the so-called “nopales” and their fruits called tunas (*Opuntia spp*.), among other plants with minor importance. For example, some species of the genus *Opuntia* have been used as the food of high nutritional importance [6,7,8,9,10]. Anticancer, antioxidant, anti-inflammatory, antiviral, antidiabetic, among other bioactivities, have been reported, from both stem and fruit extracts of these species, which demonstrated good effectiveness in different in vitro and in vivo models (reviewed in [11]).

Of the Mexican cacti, columnar species have also occupied an essential place in ethnobotany, and some of them have been used for various purposes, including medicinal, among others [2,3,12]. Compounds have been isolated from some cacti species that were active in models of inflammation induced by chemical agents such as 12-*O*-tetradecanoylphorbol 13-acetate (TPA), as well as inhibitors of the proliferation of tumor cells in cultures in vitro [13], concisely reviewed by Harlev et al. in 2013 [14]. Although the biological effects of compounds isolated from Cactaceae have been reported, their mechanisms and molecular targets are not always discussed. Many of the molecules, like sterols or triterpenes, display a wide range of biological activities, but it is necessary to explore how they act in biological systems. Our group has decided to explore the molecular mechanisms of natural product bioactivities through an in silico bioprospection approach.

Computational chemistry tools have boosted traditional natural product research. Among the strategies that have been used, Docking-Based Virtual Screening (DBVS) [15,16] has given relevant results. Though it has some critical limitations, DBVS has demonstrated to be a useful strategy to suggest the action mechanism or for proposing new biological activities of known products or extracts [17,18,19,20,21,22,23,24,25]. The selection of the bio targets to be analyzed is not always an easy feature as could be biased by the research group. Then, it is essential to carry out a profound review of the chemistry and experimentally demonstrated bioactivities of extracts and isolated compounds, and from this information, choose the most relevant bio targets for the in silico study.

Our group has dedicated for the last fifteen years, to perform systematic studies about the chemistry and biological activities of cacti [26,27,28,29,30,31,32,33]. Thus, this work presents a review of the chemistry and bioactivities of sterols and triterpenes found in Mexican Columnar Cactaceae, and in the last section, from the collected information about the chemistry and biological activities displayed from those, we selected some bioactivities and their molecular targets to be explored in the DBVS, intending to perform the in silico bioprospection of compounds found in Mexican Columnar cacti.

## 2. Chemistry of Cactaceae

The chemistry of the Cactaceae Family has been described intensively over the years. The first report about the biological effects and chemistry of a cactus was made early in the second part of eighteen century [34], which describes the use of *Cereus grandiflorus* (Linnaeus) Mill (nowadays the species is classified as *Selenicereus grandiflorus* (Linnaeus) Britton & Rose, [3,35,36]), for the treatment of certain heart diseases. The above report was described in one of the first reviews of Cactaceae chemistry [37]. Although the alkaloids and polyphenols have already been described in several species, their biological activities have been studied extensively, and a recent report has been published [12]. However, the systematic revision of the chemistry of sterols and triterpenes from cacti and their biological activities is pending. Due to the length of the topic, this review covers only aspects related to the chemistry and biological activities of sterols and triterpenes that have been isolated from Mexican species of the subfamily Cactoideae, tribe Phyllocacteae, mainly from the subtribe Pachycereeae [3,4], also named Echinocereinae [38], which is recognized as a largely North and Central American taxon. Some exceptions are made for sterols and triterpenes isolated from species belonging to other tribes or subfamilies, the unique characteristics of which are worth mentioning. The chemistry and biological activities of other chemical groups like alkaloids or betalain-pigments are not reviewed in this opportunity.

### 2.1. Sterols from Mexican Columnar Cactaceae

There is not enough data to generalize the type of the preferred sterol skeleton, the substitution pattern, and the quantities of sterols present in the different species, genera, clade or subfamily of Cactaceae. With an exception of some Sonoran Desert species (*Carnegiea gigantea*, *Pachycereus pringlei*, *Machaerocereus gummosus*, *Lemaireocereus thurberi*, among others) whose chemistry has been described exhaustively because of its ecological implications in the Cactus-Microorganism-*Drosophila* Model System of the Sonoran Desert [39,40,41]. The structures of sterols isolated from Mexican Cactaceae are presented in Table 1.

Cactaceae is a rich source of sterols, many of them with some special features (e.g., the presence of extra methyl groups at C4 or C14, or the positions of double bonds in cholesterol skeleton). The isolation of the sterols from Cactaceae began early with lophenol, schottenol, 24-methylene-lophenol, lathosterol, 5α-campest-7-en-3β-ol, spinasterol, together with locereol, and 5α-cholesta-8, 14-dien-3β-ol, which were isolated from aerial parts of *Lophocereus schotii* [42,43]. In a subsequent study, isolation of viperidinone and viperidone was reported, and deoxiviperidone from *Wilcoxia viperina* [44,45], species now called *Peniocereus viperinus* [46].

In other studies, from roots of *Peniocereus fosterianus* and *P. macdougallii* were isolated two 3β, 6α-dihydroxysterol: peniocerol and macdougallin respectively, together with small amounts of lophenol and campesterol [45,47,48]. Special mention is made for macdougallin, which is a very particular 14α-methylcholesterol that will be discussed later.

Besides, from the root of *P. greggii* was performed an investigation to find other 14α-methylcholesterols similar to macdougallin, but the authors were not successful [49]. However, from this species, they isolated deoxiviperidone, peniocerol, viperidone, viperidinone, together with a new molecule in the form of its acetylated derivative, which presented the unusual *cis*-fusion between rings A and B of the cholesterol skeleton, which they called 5β-desoxyviperidone.

On the other hand, small amounts of cholesterol were isolated from *Stenocereus thurberi*, some common sterols like campesterol and β-sitosterol [50], together with peniocerol, macdougallin, and three new 3β,6α-dihydroxysterols, named cyclostenol, stenocereol, and thurberol, the last two with the characteristic double bond in C-8 like peniocerol [51].

In another study, Jiang et al. isolated a new sterol from the aerial parts of *Opuntia dillenii*, which they called opuntisterol. Although the genera *Opuntia* does not belong to columnar Cactaceae, opuntisterol and other compounds present in *Opuntia spp*. were included in the present review because of the special feature of the configuration 5β in a sterol configuration, an infrequent feature in cacti sterols [52], but common in ecdysteroids and related phytoecdysteroids [53,54].

Finally, from the pollen of *Carnegiea gigantea*, was found 24-methylene-cholesterol, while 24-dehydropollinastanol, fucosterol, among other sterols, were found in *O. phaeacantha* and *O. versicolor*.

With these data together with that previously reported [55], it can be inferred that the sterols found most frequently in the Cactaceae family are the typical C29 and C28 compounds, with the common Δ^5^ unsaturations such as β-sitosterol, campesterol or stigmasterol. However, a distinctive feature of some species is the presence of C27 sterols, with Δ^8^ unsaturations, with an extra 6α-hydroxyl group as peniocerol. Another one distinctive feature of some species is the presence of compounds considered intermediaries in the “normal” biosynthetic pathways as lophenol or macdougallin, which strongly suggests that these plants possess modified metabolic pathways [43,45,56]. On the other hand, the presence of some sterols with unusual *cis* fusion in the joint of rings A/B like 5β-desoxyviperidone and opuntisterol, which presents the same configuration like ecdysone (the molting hormone in arthropods) could be relevant to the chemical defensive mechanisms in Cactaceae, as was previously hypothesized [27]. The species and the isolated sterols, together with the bioactivities of those compounds, are showed in Table 2 and Table 3, respectively.

### 2.2. Triterpenes from Mexican Columnar Cactaceae

One of the best-studied chemical groups of cacti is the triterpenes since they constitute, in many cases, the most abundant compounds found in Cactaceae. The triterpenes display a wide diversity of structural features, with characteristic oxidation patterns, and a particular distribution in the species, reasons why they are used for chemotaxonomic studies [94]. In addition to the above, many of the triterpenes isolated from these plants present biological activities ranging from their ecological effects [40], as well as effects on models of inflammation and anti-nociception [13,26,95], which makes their research attractive. Information of structures with an emphasis on the origin of the skeleton of each compound isolated from Cactaceae is presented in Table 4, Table 5 and Table 6. In Table 4, pentacyclic triterpenes cycloartenol, 24-methylenecycloartenol and 25(27)-dehydrolanost-8-enol are described, while lupane- and oleanane-type triterpenes are described in Table 5 and Table 6 respectively.

Although only Mexican columnar cacti were considered to the construction of the present review, a special mention is necessary to describe the triterpene content of *Trichocereus pachanoi*, a cactus plant from South America, which is an important source of some compounds named pachanols A-D (Table 7). These compounds possess a particular type of triterpene skeleton, called pachanane, characterized by the presence of five six-membered rings, and the position of the 27α-CH_3_ attached to C-15 [96,97].

Additionally, for the construction of the present review, only the aglycones of triterpenes were considered. In the first reports regarding the isolation and elucidation of those compounds, the routine procedure began with the hydrolysis of the organic extracts, isolating only the aglycones. The pattern of glycosylation of the natural compounds, and the number and relative positions of sugar moieties should be discussed in another review. Specific information about the content of triterpenes by species, as well as their biological activities, are described in Table 8 and Table 9, respectively.

It should be noted that of the following genera listed in Mexico: *Acanthocereus*, *Bergerocactus*, *Cephalocereus*, *Echinocereus,* and *Neobuxbaumia*, have not been revised for their sterol nor triterpenoid content, so the chemistry of those species will have to be explored, to generate knowledge about the kind of natural products presents in each genus and their bioactivities.

From this review, it is evident that sterols and triterpenes from *Cactaceae* exhibit a myriad of bioactivities, mainly anti-inflammatory, metabolic regulatory, and CNS protective activities standing out. The lack of information about the molecular mechanism displayed of sterols and terpenoids prompt us to perform an in silico bioprospection in some molecular targets. Among the targets that have been commonly assayed against each biological process, only COX-1, COX-2, PTP1B, PPAR-α, PPAR-γ, acetylcholinesterase (AChE), LXR-α and LXR-β were selected for the in silico study to explore the theoretical molecular affinity of sterols and triterpenoids against each target. Then, the information presented in the results section, do not represent a validation of the molecular mechanism in the case of good molecular coupling between the ligands and the targets, nor do they represent the verification of the null activity of the ligands in the molecular targets. The data from molecular docking only represents a guide to future research designed to discover the specific molecular mechanisms that each molecule presents in biological models.

## 3. In Silico Bioprospection of Bioactive Compounds

All sterols and triterpenes described in this review were constructed as ligands using Chem Sketch from Advanced Chemistry Development [202] and exported to Spartan ’10 as mol files. The geometry of all molecules was optimized using MMFF//HF 6-31 G*, and final structures were included in an SDF file. The docking studies were carried out using Molegro Virtual Docker v.6.0.1 [203] based on the crystal structures of some bio targets that have been proposed for the biological activities of *Cactacaeae* extracts or purified compounds derived from them: (a) anti-inflammatory activity—cyclooxygenase-1 (COX-1, PDB code: 1Q4G [204]) and cyclooxygenase-2 (COX-2, PDB code: 3NT1 [205]); (b) metabolic activity—PPAR-α (PDB code: 2ZNN [206]), PPAR-γ (PDB code: 5Y2T [82]) and PTP1B (PDB code: 1C83 [207]); (c) neuroprotective activity—LXR-α (PDB code: 3IPU [208]) LXR-β (PDB code: 1P8D [209]) and acetylcholinesterase (PDB code: 4EY6 [210]). All 3D protein structures were retrieved from the Protein Data Bank [211]. Docking studies were carried out using a previously reported methodology [17,18]. Briefly, all the solvent molecules and cocrystallized ligands were removed from the downloaded structures. The active sites of each enzyme or the ligand-binding domain (LBD) were chosen as the searching sites centered on the cocrystallized ligand, except for PTP1B where the docking area was centered on allosteric site B as previously described [212], and delimited with a 15 Å radius sphere. Standard software procedure was used using the *MolOptimizer* algorithm. The assignments of charges on each protein were based on standard templates; no other charges were necessary to be set. The Root Mean Square Deviation (RMSD) threshold for multiple cluster poses was set to <2.00 Å. The docking algorithm was set to 5000 maximum iterations with a simplex evolution population size of 50 and 25 runs for each ligand. After docking, *MolDock Score* was calculated as the theoretical binding affinity, lower values of the score are related to better binding. For each ligand, the pose with the lowest score was selected for further analysis. Cocrystallized ligands were also docked to their respective receptors to verify the efficacy of this procedure, the top-ranking score was recorded, and the RMSD of that pose from the PDB original structure was computed. In all the cases, the RMSD values were lower than 2.5 Å.

### 3.1. Antiinflammatory Molecular Targets

Table 10 and Table 11 show the results obtained for the docking study carried on COX-1 and COX-2. In both tables, the top-10 compounds with higher affinity are displayed, but a complete table of all the results is included as part of Supplementary information. Table 10 shows the data from sterol derivatives, and Table 11 shows the data from triterpene derivatives that had lower MolDock scores.

**Figure 1:**
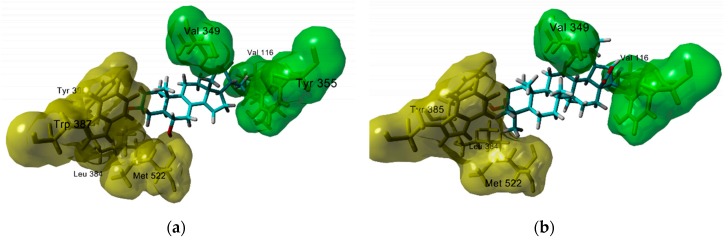
Docking poses of (**a**) peniocerol, a sterol, and (**b**) 22-hydroxystellatogenine, a triterpene with COX-1.

The analysis of the binding mode of the sterols with higher affinity reveals some structural features that improve ligand binding. In the first instance, among sterols, the oxidation pattern at C3, C6, and C7 appears to be relevant for the interaction with residues Leu 384, Tyr 385, Trp 387 and Met 522 in both COX enzymes. Notably, a hydrogen bond interaction to Met 522 is constant in all 6-OH substituted derivatives like peniocerol. Interestingly, a small hydrophobic pocket is located between these residues (in yellow in Figure 1a) and is occupied by the methyl group in position 4 of locereol, slightly increasing theoretical affinity. Other critical structural factors for enzyme binding are the unsaturations in rings B and D. The unsaturation in ring B seems to be necessary for optimal interaction of substituents in position 6 and potentially 7 with Met 522. Whereas, unsaturation in ring D is needed for accommodation of the aliphatic chain in a hydrophobic pocket formed by Val 116, Val 349, and Tyr 355 (for example, thurberol has better theoretical affinity than peniocerol). Additional substituents in the aliphatic chain improve enzyme binding, as compounds with additional methylene or ethylene groups have slightly more affinity than their analogs than do not bear these groups. (i.e., 24-methylenelophenol has more affinity than lophenol).

On the other hand, the analysis of the interaction of triterpenes shows that they could similarly bind to COX-1 and COX-2 as sterols. Interestingly, the first 9 compounds with better theoretical affinities belong to the lupane skeleton. In general, the ring A of all compounds interacts with Leu 384, Tyr 385, Trp 387, and Met 522 in the same fashion as sterols (in yellow in Figure 1a,b). The 4,4-dimethyl group in ring A occupies the small hydrophobic group ubicated between these residues. However, it seems to be too large to fit on this site, leading to a lower theoretical affinity. The most oxidized rings (usually rings D and E) in 22β-hydroxystellatogenin interact with the same residues that the side chain of sterol does. The interaction is through hydrogen bonding to residues Ser 353, Tyr 349, and Try 355 of COX-1 or Tyr 348 and Try 385 in COX-2 (in green in Figure 1b). Although hydroxylation in rings D and E improves theoretical affinity, the number of hydroxy or keto groups is not correlated with theoretical affinity, suggesting that hydrophobic interaction is more critical of ligand binding.

COX-2 is one of the preferred molecular targets of NSAIDs [17]. The literature is full of research papers about the design, synthesis or isolation together with in silico, in vivo and ex vivo studies to the development of new anti-inflammatory drugs. Although the clinical importance of selectivity against COX-2 vs COX-1 was earlier discussed [213], many groups focus their research programs to discover selective COX-2 drugs. The top ten sterols and triterpenes with the highest MolDock scores cannot be considered selective against any COX enzyme if we consider the MolDock Score as a measure of the theoretical affinity of the compound. From the top 10 ligands with better MolDock score, fucosterol, spinasterol, 24-methylenecholesterol, β-sitosterol, and peniocerol in the sterols group and lupeone and lupeol of triterpene group are well known anti-inflammatory molecules, with mechanisms including COX-2 inhibitory activities. It is possible to focus the next survey to isolate and determine the anti-inflammatory activities and their molecular mechanisms of compounds like thurberol, locereol or lophenol, together with thurberogenine, betulinic aldehyde, 16β- and 22β-hydroxystellatogenin or machaerogenine which anti-inflammatory activities or their inhibitory activities against COX enzymes are still unknown.

### 3.2. Antidiabetic and Metabolic Activities

Several studies have positioned both sterols and triterpenes as hits for the development of drugs for the treatment of metabolic diseases, including diabetes mellitus type 2 [214,215]. Table 12 shows the top 10 sterol ligands, which exhibited the highest theoretical affinity for PTP1B, PPAR-α, and PPAR-γ. Ligands showed in Table 12 exhibited the highest affinity for all the three bio targets and could be considered as potential multitarget molecules for the management of complex metabolic diseases like metabolic syndrome. However, other sterols not shown in this Table exhibited a good affinity for two of the targets like lophenol, locereol, and 5a-cholesta-8,14-dien-3β-ol, which had good theoretical affinity against PPAR-α and PPAR-γ, some synthetic ligands have been developed as PPARα/γ dual agonists to achieve a broad spectrum of metabolic effects with actions against dyslipidemia and hyperglycemia. Deoxyviperidone, 5β-deoxyviperidone, and lathosterol had good scores against PTP1B and PPAR-γ, which could be a good combination of bio targets for the treatment of diabetes mellitus type 2. Well known antidiabetic sterols like fucosterol, lophenol and β-sitosterol have demonstrated in several studies their potential to regulate at different levels both PTP1B and/or PPAR proteins [61,216,217]. Although lophenol does not belong to the top 10 molecules with higher theoretical affinity, the Moldock score values for both PPAR-α and PPAR-γ proteins were -130.9 and -129.7, suggesting that the methodology used correctly predicts the biological activity. Special attention should be taken with sterols like schottenol, spinasterol, thurberol cyclostenol 24-methylenecholesterol, peniocerol, among other sterols that were considered as good candidates to future antidiabetic research.

In the case of triterpenes (Table 13), the results strongly suggest that the hydroxylation pattern in rings D and E seems important for enzyme binding. Notably, it is remarkable that many of the triterpene derivatives bear a 17-carboxylic group.

**Figure 2:**
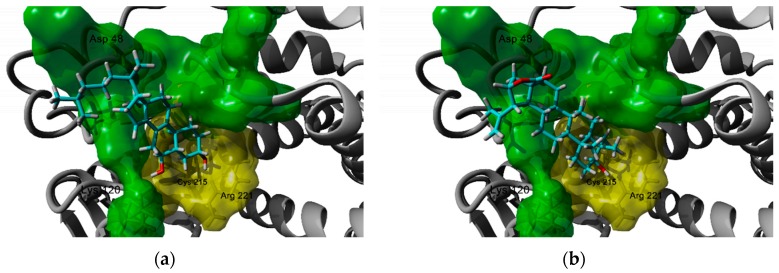
Docking poses of (**a**) peniocerol, a sterol, and (**b**) 22-hydroxystellatogenin, a triterpene with PTP1B. In yellow, the active catalytic site, in green the allosteric site B.

Analysis of the predicted complexes show that despite the searching area was centered on the close allosteric site B, which is comprised by residues Arg 24, Arg 254, Glu 262, Tyr 46, Asp 48, Val 49, Ile 219, and Met 258 [218], both sterols and triterpenes can interact with this site but also with the catalytic site, particularly with two of the most critical residues Cys 215 and Arg 221. This finding could explain the non-competitive and mixed-inhibition properties of some compounds, which has been experimentally demonstrated [212,219]. For example, fucosterol and β-sitosterol mainly occupy allosteric site B, while 6-hydroxy substituted sterols bind to both the catalytic site (via the 3β-OH group, the 6α-OH interacts through a hydrogen bond to Tyr 46 and Lys 120) and the allosteric site (Figure 2a). In the case of triterpenes, some of the derivatives with better affinity have hydroxy or carboxylic groups in rings D and E which can interact with amino acids located in site B like Arg 47 (for 17-COOH substituted derivatives) and Asp 48 (for lactone derivatives as shown in Figure 2b).

**Figure 3:**
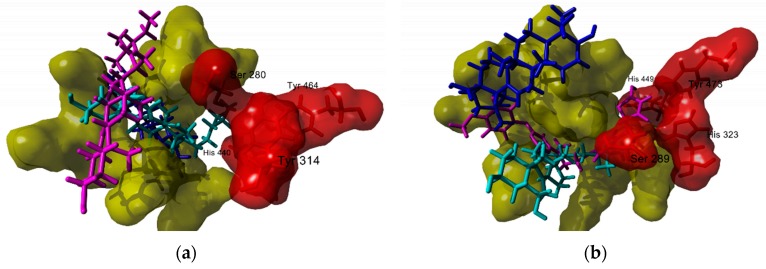
(**a**) Docking poses of selected sterols and triterpenes in the ligand-binding domain (LBD) of PPAR-α, shown in yellow and red for the acid-binding site. Three different modes of potential binding to PPAR-α were suggested in the docking study, as exemplified with peniocerol (in cyan), oleanolic acid (in blue), and schottenol (in magenta); (**b**) A similar situation was found for PPAR-γ, with two potential binding modes. The LBD site is shown in yellow and red for the thiazolidinedione binding site. Peniocerol is shown in cyan, oleanolic acid in blue, and known agonist lobeglitazone in magenta is included for comparison purposes.

Analysis of the predicted poses of the testes compounds to PPAR-α revealed three potential binding modes (Figure 3): (a) sterols with no hydroxyl groups in ring B interact in a cavity distinct to the one occupied by known synthetic partial agonists; (b) 6-hydroxy substituted sterols (like thurberol and peniocerol) and lactone bearing triterpenes (like stellatogenin) bind to the same site of synthetic agonists; and (c) acidic triterpenes occupy the same site as known partial agonists, with the carboxyl group approximating to the same residues that interact with the carboxyl group present in the majority of PPAR partial agonists. This mode of interaction could explain the demonstrated activity of oleanolic acid as a PPAR-α activator [220]. For the PPAR-γ docking study, two different modes of binding were found. The first one was common for most triterpenes, these compounds bounded in a different site than that of known synthetic agonists, away from the thiazolidinedione (TZD) binding site formed by residues Ser 289, His 323, His 449 and Tyr 473. The second one was common for sterols; the tetracyclic skeleton occupied an alternative pocket, and the lateral chain could locate either inside or outside of the TZD binding site (Figure 3b). It would be expected that these alternative modes of binding in both nuclear receptors lead to none or different levels of activation. Thus, additional studies should be carried out.

Some triterpenes from Cactaceae like β-amyrin, oleanolic aid, oleanolic aldehyde, lupeol, betulin, betulinic acid, lupeone and cycloartenol which are well known to regulate some molecular targets of diabetes pathology, with a good prediction in this review. We need to explore the antidiabetic activities, particularly the activity against molecular targets involved in metabolic diseases, of some triterpenes like 16β-hydroxystellatogenin, thurberogenin, stellatogenin, myrtillogenic acid, alamosogenin, 22β-hydroxystellatogenin, machaeric acid, and machaerinic acid, all of them characteristic items of the Cactaceae chemistry, which display some specific oxidation patterns as was discussed above.

### 3.3. Neuroprotective Activity

As life expectancy has increased, neurodegenerative diseases have become a growing concern, and the need for efficient treatments has become an urgency. Acetylcholinesterase inhibitors have been used for the management of Alzheimer’s disease, and some have suggested their neuroprotective potential. Also, agonists of LXR-α and LXR-β have been studied for their potential use as neuroprotectors. Then, the development of agonists of these receptors that could also act as AChE inhibitors could lead to interesting treatments. Table 14 and Table 15, respectively, show the sterols and triterpenes ligands with a higher affinity to the previously mentioned targets.

**Figure 4:**
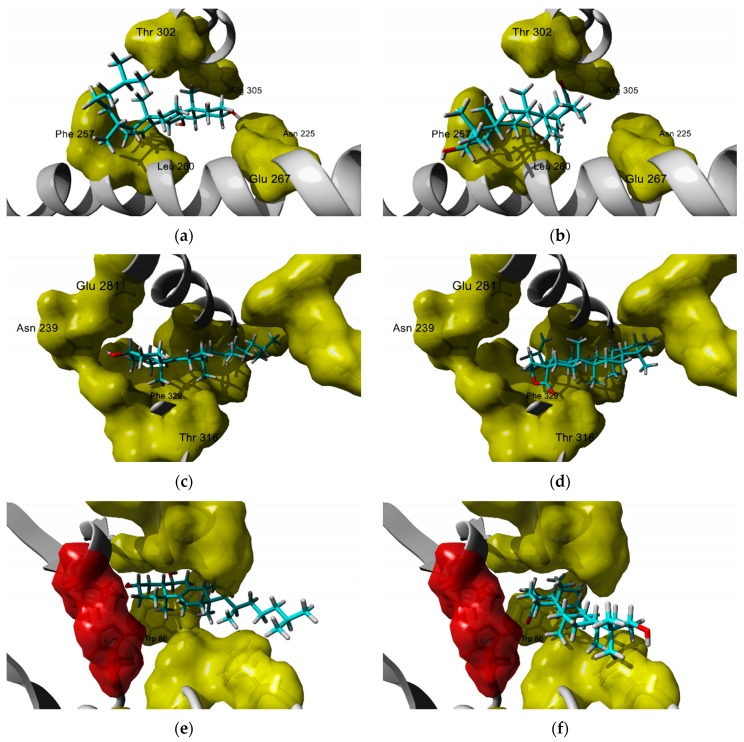
Predicted poses for selected sterol (thurberol) and triterpene (thurberogenin) to some bio targets related to neuroprotection. (**a**) Thurberol—LXR-α; (**b**) thurberogenine—LXR-α; (**c**) thurberol—LXR-β; (**d**) thurberogenine—LXR-β; (**e**) thurberol—AChE; (**f**) thurberogenine—AChE. Residues relevant for interactions through hydrogen bonding are labeled.

For LXR-α, it was found that both sterols and triterpenes bind to the same site (Figure 4a) but with different interaction patterns. Sterols bind through hydrogen bonding via the 3β-OH group to Glu 267 and Asn 225 while the side alkyl chain interacts with residues Phe 257, Leu 260, Thr 302, and Phe 315. In general, oxidation of ring B improves ligand binding, and slight differences in docking score can be attributed to the nature of the alkyl chain; theoretical affinity increased with the incorporation of additional methylene groups, probably through new hydrophobic interactions. Triterpene derivatives interact with residues Thr 302 and Arg 305 via hydrogen bonding to the lactone or the 17-COOH group of rings D and E. The absence of hydroxy groups in these rings slightly diminishes the theoretical affinity.

The docking score calculated for the predicted complexes of the analyzed sterols and triterpenes is close to the score for the known LXR-β ligand 24(*S*),25-epoxycholesterol (MolDock score = 151.3). The interaction of the 3β-OH group to Asn 239, Glu 281, and Arg 319 are constant in all derivatives (Figure 4c). As it was the case of LXR-α docking study, oxidation in position 6 improves theoretical affinity through interaction to Phe 243, and Phe 329 and additional differences in docking score among sterols can be accredited to the nature of the alkyl chain side. Whereas triterpenes interact through hydrogen bonding to residue Thr 316 via oxygen atoms of lactone ring or 17-COOH, this interaction pattern was common in most of the derivatives analyzed. Interestingly, ring A lays close to residues His 435 and Trp 457, which interact with the epoxy group of 24(*S*),25-epoxycholesterol, and could interact with hydroxyl groups of 22-hydroxycholesterol and 24-hydroxycholesterol, known endogenous ligands of LXRs. It has been reported that oxidation at both C-22 and C-24 increases LXR activation [221].

According to the results, for the interaction of triterpenes with the enzyme acetylcholinesterase, the substitution pattern in ring A is essential, since that ring A is the one that can approximate the catalytic site. On the other hand, in the B ring, the presence of a hydroxyl group in the C6 helps due to the interaction of the oxygen in the hydroxyl moiety with the Trp 86 located in the protein gorge. In the same set of interactions shown by these compounds, the side chain of cholesterol is stuck with the outer part (mouth) of the enzyme. In triterpenes, the E ring is the one that interacts with the catalytic site through interactions of C17-COOH or lactone moieties, with some residues in the active site. The absence of these features diminishes slightly the affinity. On the other hand, the H atom of 3β-OH of the triterpenes interacts with Tyr 341. In this review is evident the potential that some sterols and triterpenoids from Cactaceae, to the development of new neuroprotectors, via the inhibition of acetylcholinesterase and LXR-α and LXR-β.

## 4. Conclusions

The oxidation pattern in sterols and triterpenes from Cactaceae is an extraordinary feature. The oxidation at C6 to form a 6α-hydroxyl group in cholesterol moiety in sterols like peniocerol, macdougallin, cyclostenol, stenocereol, and thurberol, as well as the C6 ketone in viperidinone, viperidone, deoxyviperidone, and 5β-deoxyviperidone, appears to be essential to explaining the possible molecular mechanisms behind the biological activities of those kinds of compounds, as suggested by the available experimental bioactivities and the results of the present in silico study. On the other hand, the oxidation pattern in E ring in oleanane and lupane triterpenes appears as a pivotal factor in controlling the affinity of compounds. At least in the in vitro assays, the presence of hydroxyl, ketone, carboxylic acid, or lactone groups (thurberogenin and 16β-hydroxystelltogenin) in ring E appears to be important in the selectivity of the sterols and triterpenes with their preferred target.

The chemistry of sterols and triterpenoids of some species, from the subfamily Cactoideae, tribe Phyllocacteae, mainly from the subtribe Echinocereinae, was reviewed, together with their biological activities. The information generated about the sterols reveals the presence of the typical △^5^ sterols like sitosterol, among others, but interestingly the presence of a very unusual family of △^8^ sterols with a pattern of oxidation in a sterol moiety of 27 carbons. Two sterols are of biosynthetic importance because it appears as unusual intermediaries in a truncated demethylation process. On the other hand, triterpenes appear as common compounds with unique features, including the oxidation pattern in rings C/D. The compounds found in Cactaceae have been extensively studied because of their biological properties. In this paper, we focused only on three biological activities. At least at an in silico level, it is possible to correlate the biological activities with the theoretical affinities showed between the compounds and some of the specific receptors involved as molecular targets of chronic diseases like inflammation, type 2 diabetes or neurodegenerative disorders. Regarding the in silico bioprospection, this study reveals that the oxidation pattern in ring B of sterol skeleton and rings D and E of triterpenes, together with the presence of lactones, contribute to the biological activities of Cactaceae triterpenes. The above prompt us to continue with this methodological approach to find highly bioactive hits from the sterols and triterpene of Cactaceae, useful for the development of semisynthetic drugs for the management of chronic degenerative diseases with a multitarget approach.

## Figures and Tables

**Table 1:** Chemical structures of sterols isolated from Cactaceae.

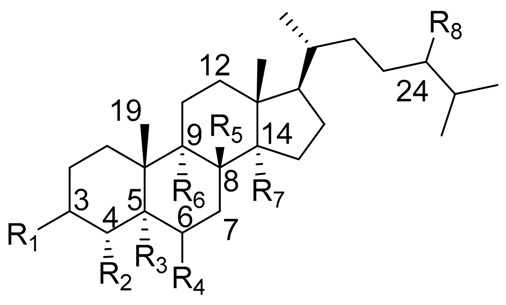									
Sterol Name	R_1_	R_2_	R_3_	R_4_	R_5_	R_6_	R_7_	R_8_	△^1^
Cholesterol	β-OH	H	-	-	H	H	H	H	5
Lophenol	β-OH	CH_3_	H	H	-	H	H	H	7
Schottenol	β-OH	H	H	H	-	H	H	β-CH_2_CH_3_	7
24-methylenelophenol	β-OH	CH_3_	H	H	-	H	H	=CH_2_	7
Lathosterol	β-OH	H	H	H	-	H	H	H	7
5α-campest-7-en-3 β-ol	β-OH	H	H	H	-	H	H	β-CH_3_	7
Spinasterol	β-OH	H	H	H	-	H	H	β-CH_2_CH_3_	7
Locereol	β-OH	CH_3_	H	H	-	-	-	H	8, 14
5α-cholesta-8,14-dien-3β-ol	β-OH	H	H	H	-	-	-	H	8, 14
Viperidinone	β-OH	H	H	=O	-	α-OH	OH	H	7
Viperidone	β-OH	H	H	=O	-	α-OH	H	H	7
Deoxiviperidone	β-OH	H	H	=O	-	H	H	H	7
Peniocerol	β-OH	H	H	α-OH	-	-	H	H	8
Macdougallin	β-OH	H	H	α-OH	-	-	CH_3_	H	8
5β-deoxiviperidone	β-OH	H	β-H *	=O	-	H	H	H	7
Cyclostenol **	β-OH	H	H	α-OH	H	-	CH_3_	H	-
Stenocereol	β-OH	H	H	α-OH	-	-	CH_3_	H	8,24(25)
Thurberol	β-OH	H	H	α-OH	-	-	-	H	8, 14
Opuntisterol ***	H	H	β-H *	β-OH	H	-	H	β-CH_2_CH_3_	9
24-methylenecholesterol	β-OH	H	-	-	H	H	H	=CH_2_	5,24(28)
24-dehydropollinasterol **	β-OH	H	H	H	H	-	CH_3_	H	24(25)
Fucosterol	β-OH	H	-	-	H	H	H	=CH-CH_3_	5,24(28)
β-Sitosterol	β-OH	H	-	-	H	H	H	β-CH_2_CH_3_	5

* Both sterols, 5β-deoxiviperidone and opuntisterol present the *cis* configuration in rings A–B fusion. ** Cyclostenol and 24-dehydropollinasterol presents a β-cyclopropane moiety at C19–C9. *** Opuntisterol special feature is the presence of a 12α-OH. **^1^** The specific position of the double bond of each compound is defined by the absence of the respective atom or atoms in carbon positions, and the position of the double bond designed in the column with the symbol **∆**.

**Table 2:** List of species and the isolated sterols.

Genus	Specie	Compound	Isoltated from ^1^	Reference
*Lophocereus*	*L. schotti* (Engelm.) Britton & Rose	Lophenol	AP	[42,43]
		Schottenol		
		24-methylenelophenol		
		Lathosterol		
		5α-campest-7-ene-3β-ol		
		Spinasterol		
		Locereol		
		5α-cholesta-7,14-dien-3β-ol		
*Leptocereus*	*L. quadricostatus* (Bello) Britton & Rose	Viperidone	AP	[57]
*Peniocereus*	*P. viperinus* (F.A.C. Weber) Buxb.	Viperidinone	R	[44]
		Viperidone		
		Deoxiviperidone		
	*P. fosterianus* (Cutak) Lodé	Peniocerol	R	[47]
	*P. macdugalli* Cutak	Lophenol	R	[45]
		Peniocerol		
		Macdougallin		
		5β-desoxyviperidone		
	*P. greggi* (Engelm.) Britton & Rose	5β-desoxyviperidone	R	[49]
*Myrtillocactus*	*M. geometrizans* (Mart. ex Pfeiff.) Console	Peniocerol	R	[26,33]
		Macdougallin		
*Stenocereus*	*S. thurberi* (Engelm.) Buxb.	Cyclostenol	AP	[51]
		Stenocereol		
		Thurberol		
	*S. stellatus* (Pfeiff.) Riccob.	β-sitosterol	AP	[32]
*Opuntia* *	*O. dillenii* (Ker Gawl.) Haw.	Opuntisterol	AP	[52]
	*O. phaeacantha* Engelm.	24-methylenecholesterol	P	[58]
	*O. versicolor* **	Pollinastanol	P	
		24-methylenecholesterol		
		Fucosterol		
*Carnegiea*	*C. gigantea* (Engelm.) Britton & Rose	24-methylenecholesterol 24-dehydropollinastanol	P	[59,60]
*Pachycereus*	*P. pringlei* (Watson) Britton & Rose	24-methylenecholesterol	P	[58]

* Although *Opuntia* is not in the Pachycereeae tribe (Opuntieae, Opuntioideae, Cactaceae), because of the diversity and special conformation in one of their sterols, we decide to include it in this review. ** O. versicolor is a synonimus of *Cylindropuntia versicolor* (Engelm. ex J.M.Coulter) F.M.Knuth. ^1^ AP: aerial parts; R: roots; P: pollen.

**Table 3:** Bioactivities of selected Cactaceae sterols.

Compound	Activity	Description	Reference
Lophenol	AD, CT	AD = Agonist of PPARα and PPARγ, changing the expression of genes involved in fatty acid transport, binding and oxidation in mouse liver. CT = Moderate cytotoxic effect against the L5178Y-R cell line.	[61,62,63]
Schottenol	MM	LXR agonists modulating gene expression of LXRα and LXRβ liven nuclear receptors.	[64]
Lathosterol	AM and CT	AM = Anti-mutagenic activity against MNNG and NQO. CT = Moderate cytotoxic effect was shown by the compound against MES-SA, MCF-7, and HK-2 cell lines.	[65,66]
Spinasterol	AI, AN, CR, CT and MM	AI = Inhibitory activity against COX-1 and COX-2 enzymes and antagonistic effect on the TRPV1 receptor. AN = Inhibitory activity against *Helicobacter pylori*CR = Antidepressant-like effect due to the regulation of the TRPV1 receptor. CT = Moderate cytotoxic effect against HeLa, MCF-7, MDA-MB-231, and SKOV-3 cell lines by inducing G0/G1 arrest stimulating the expression of p53 and Bax genes and lower expression of cdk4/6 genes. MM = LXR agonists modulating gene expression of LXRα and LXRβ liven nuclear receptors.	[64,67,68,69,70,71,72,73]
Viperidone	MM	Strong inhibition binding to LXRα with an IC_50_ value of 0.10 μM.	[57]
Peniocerol	AI, CT and IN	AI = Potent edema inhibition in TPA induced edema assay. CT = inhibition of breast and colon carcinoma MCF-7 and HCT-15 cell lines proliferation through cell cycle arrest and apoptosis in both cell lines. Also, peniocerol causes Mitochondrial permeability transition (MPT) induction. IN = insect growth regulatory activity against *Spodoptera frugiperda* and *Tenebrio molitor*.	[26,33,74,75]
Macdougallin	AI, CT and IN	AI = moderate edema inhibition in TPA induced edema assay. CT = inhibition of leukemia K-562 cell line proliferation. IN = insect growth regulatory activity against *Spodoptera frugiperda* and *Tenebrio molitor*.	
24-dehydropollinastanol	AD	Agonist of PPAR-α and PPAR-γ, changing the expression of genes involved in fatty acid transport, binding and oxidation in mouse liver.	[61,62]
24-methylenecholesterol	AI, AN, and CT	AI = Low inhibition of key inflammatory enzymes like COX and NF-κB1. AN = Inhibitory effect for *Trypanosoma brucei brucei* and *Mycobacterium marinum*. CT = Potent inhibition of aromatase which is a therapeutic target for breast cancer treatment and has a cytotoxic effect on HS27 cell line.	[76,77,78]
Pollinastanol	CT	Inhibition of aromatase which is a therapeutic target for breast cancer treatment.	[77]
Fucosterol	AD, AO, AN, CR, AI, CT and HT	AD = Inhibition of sorbitol accumulation and diabetic key enzymes like RLAR, HRAR, PTP1B, and α-glucosidase. It also has a downregulation effect of PPAR-γ, C/EBPα, and SREBP1. AO = Regulate transaminase activity (sGOT, sGPT) and enhances the antioxidant activity of SOD and GSH-px. AN = High inhibitory effect against the parasite *P. falciparum*. CR = Increases serotonin and noradrenaline in the central nervous system, and it also increases central BDNF levels. Also, it showed cholinesterase inhibitory activity and neuroprotective effects.AI = Represses *i*NOS, TNF-α, and IL-6 binding to NF-κB and inhibits COX-2. CT = Induced HL-60 and HeLa cell line apoptosis through a mitochondrial pathway. HT = Inhibits the synthesis of glucocorticoid receptors involved in the regulation of ACE decreasing its levels.	[79,80,81,82,83,84,85]
β-sitosterol	AI, AD, AN, CT, MM, and IM	AI = Inhibits TNF-α, and NF-κB AD = Decreases glycated hemoglobin, serum glucose, and nitric oxide and increases insulin levels slightly. All this is a result of its potent antioxidant activity in the pancreas. AN = Growth inhibitory activity against bacteria *P. smartii* and the parasite *P. falciparum*. CT = Strong cytotoxic effect against A549 cell line by inducing apoptosis via ROS-mediated mitochondrial dysregulation. MM = Mediates cholesterol metabolism by increasing sterol excretion and decreasing cholesterol absorption and synthesis. IM = Inhibits T cell proliferation and blocks the secretion of Th2 and cytokines IL-4 and IL-10.	[86,87,88,89,90,91,92,93]

AD = Antidiabetic, AM = Antimutagenic, AO = Antioxidant, AN = Anti-infective, CR = CNS Regulation, AI = Anti-inflammatory, CT =Cytotoxic, MM = Modulation of Cholesterol metabolism, HT = Hypertension, IN = insecticidal, IM = Immunosuppressive.

**Table 4:** Cycloartane terpenes isolated from Cactaceae.

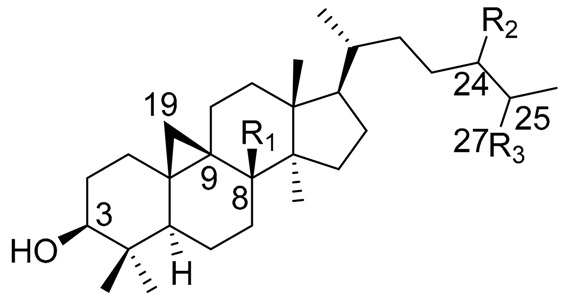				
Triterpene Name	R_1_	R_2_	R_3_	△
Cycloartenol	H	H	CH_3_	24(25)
24-methylenecycloartenol	H	=CH_2_	CH_3_	24(31)
25(27)-dehydrolanos-8-enol *	-	H	=CH_2_	8(9), 25(27)

* The cyclopropane moiety is absent instead of a C-19 β-methyl group, and the presence of a double bond between 8(9).

**Table 5:** Lupane-type triterpenes from Mexican Columnar Cactaceae.

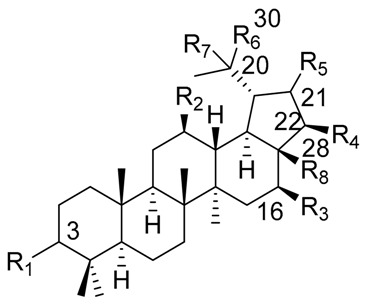									
Triterpene Name	R_1_	R_2_	R_3_	R_4_	R_5_	R_6_	R_7_	R_8_	△
Lupeol	β-OH	H	H	H	H	=CH_2_	-	CH_3_	20(30)
Betulin	β-OH	H	H	H	H	=CH_2_	-	CH_2_OH	20(30)
Betulinic acid	β-OH	H	H	H	H	=CH_2_	-	COOH	20(30)
Stellatogenin *	β-OH	H	H	H	β-O-	CH_3_	OH	CO-	-
Lupenone	=O	H	H	H	H	=CH_2_	-	CH_3_	20(30)
Thurberogenin *	β-OH	H	H	H	β-O-	=CH_2_	-	CO-	20(30)
21-ketobetulinic acid	β-OH	H	H	H	=O	=CH_2_	-	COOH	20(30)
16β-hydroxybetulinic acid	β-OH	H	OH	H	H	=CH_2_	-	COOH	20(30)
22β-hydroxystellatogenin *	β-OH	H	H	OH	β-O-	CH_3_	OH	CO-	-
16β-hydroxystellatogenin *	β-OH	H	OH	H	β-O-	CH_3_	OH	CO-	-
Calenduladiol	β-OH	OH	H	H	H	=CH_2_	-	CH_3_	20(30)
Betulinic aldehyde	β-OH	H	H	H	H	=CH_2_	-	COH	20(30)

* Stellatogenin, Thurberogenin, 22β-hydroxystellatogenin, and 16β-hydroxystellatogenin display a β-gamma-lactone between C-28 and C-21.

**Table 6:** Oleanane-type triterpenes isolated from Mexican columnar Cactaceae.

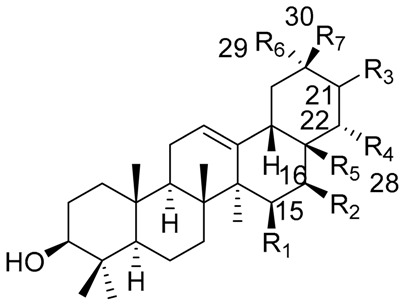							
Triterpene Name	R_1_	R_2_	R_3_	R_4_	R_5_	R_6_	R_7_
β-amyrin	H	H	H	H	CH_3_	CH_3_	CH_3_
Oleanolic acid	H	H	H	H	COOH	CH_3_	CH_3_
Oleanolic aldehyde	H	H	H	H	COH	CH_3_	CH_3_
Erytrodiol	H	H	H	H	CH_2_OH	CH_3_	CH_3_
Maniladiol	H	OH	H	H	CH_3_	CH_3_	CH_3_
Longispinogenin	H	OH	H	H	CH_2_OH	CH_3_	CH_3_
Dumortierigenin *	-O-	H	H	OH	CO-	CH_3_	CH_3_
Cochalic acid	H	OH	H	H	COOH	CH_3_	CH_3_
Myrtillogenic acid	H	OH	H	H	CH_2_OH	COOH	CH_3_
Chichipegenin	H	OH	H	OH	CH_2_OH	CH_3_	CH_3_
Olean-12-ene-3β,16β,22α-triol	H	OH	H	OH	CH_3_	CH_3_	CH_3_
Alamosogenin	H	OH	H	H	COH	CH_3_	CH_2_OH
Gummosogenin	H	OH	H	H	COH	CH_3_	CH_3_
Machaerogenin *	H	H	β-O-	H	CO-	CH_3_	CH_2_OH
Machaeric acid	H	H	=O	H	COOH	CH_3_	CH_3_
Queretaroic acid	H	H	H	H	COOH	CH_3_	CH_2_OH
Treleasegenic acid	H	H	β-OH	H	COOH	CH_3_	CH_2_OH
Machaerinic acid	H	H	β-OH	H	COOH	CH_3_	CH_3_

* Dumortierigenin and machaerogenin possess a lactone moiety at C-15, C-28, and C-21, C-28, respectively.

**Table 7:** Structures of some additional triterpenes isolated from Cactaceae.

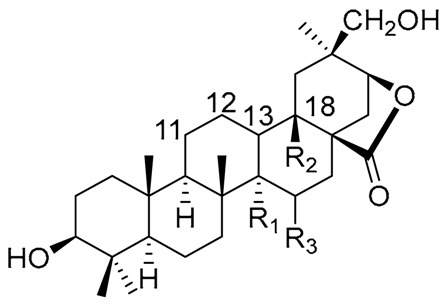	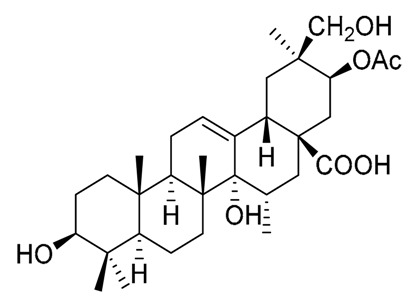	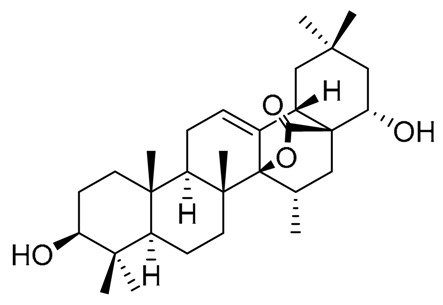
Pachanol A R_1_ = -, R_2_ = H, R_3_ = CH_3_; △^12(13), 14(15)^ Pachanol B R_1_ = H, R_2_ = -, R_3_ = αCH_3_; △^11(12), 13(18)^	Pachanol C	Pachanol D
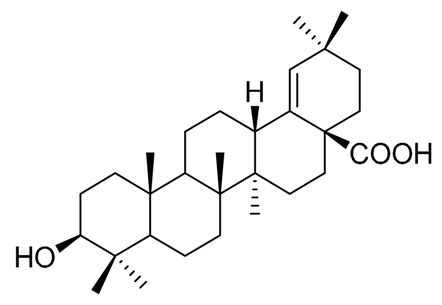	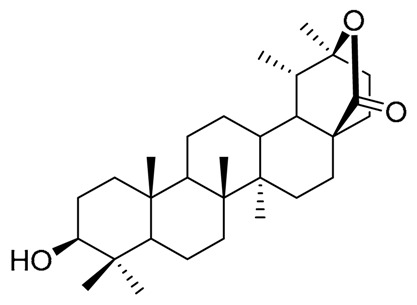	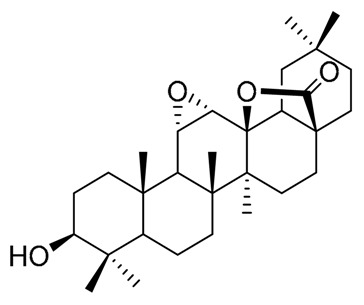
Morolic acid	27-desoxiphillirigenin	3β-hydroxy-11α,12α-epoxyolean-28,13β-olide

**Table 8:** Triterpene content by species in Mexican columnar cacti.

Genus	Specie	Compound	Isolated from ^1^	Reference
*Carnegiea*	*C. gigantea* (Engelm.) Britton & Rose	Cycloartenol, 24-methylenecycloartenol 25(27)-dehydrolanost-8-enol Lupeol	P	[60]
*Escontria*	*E. chiotilla* (Weber) Rose	Oleanolic acid	AP	[98]
		Betulin		
		Betulinic acid		
		Maniladiol		
		Erythrodiol		
		Longispinogenin		
*Isolatocereus*	*I. dumortieri* (Scheidweiler) Backerberg	Dumortierigenin	AP	[99,100,101]
		Pachanol D		
*Lophocereus*	*L. schotti* (Engelmann) Britton & Rose	Lupeol	AP	[42,43]
*Myrtillocactus*	*M. cochal* (Ocutt) Britton & Rose	Longispinogenin	AP	[102,103,104,105,106]
		Cochalic acid		
		Myrtillogenic acid		
		Chichipegenin		
	*M. geometrizans* (Mart. ex Pfeiff.) Console	Longispinogenin	AP	
		Cochalic acid		
		Myrtillogenic acid		
		Chichipegenin		
	*M. eichlamii* Britton & Rose	Oleanolic acid	AP	
		Maniladiol		
		Longispinogenin		
		Cochalic acid		
		Myrtillogenic acid		
		Chichipegenin		
	*M. schenkii* (Purpus) Britton & Rose	Oleanolic acid	AP	
		Stellatogenin		
*Pachycereus*	*P. pringlei* (Watson) Britton & Rose	Lupeol	P	[58]
		25(27)-dehydrolanost-8-enol		
	*P. weberi* (Coulter) Britton & Rose	Cochalic acid	AP	[95]
*Peniocereus*	*P. fosterianus* (Cutak) Lodé	Chichipegenin	R	[47]
	*P. macdougalli* Cutak	β-amyrin	R	[45]
*Polaskia*	*P. chende* Gibson & Horak	Oleanolic acid	AP	[107]
		Erythrodiol		
		Oleanolic aldehyde		
	*P. chichipe* (Gosselin) Backeberg	Oleanolic acid	AP	[103,108]
		Longispinogenin		
		Chichipegenin		
		Olean-12-ene-3β,16β,22α-triol		
*Stenocereus*	*S. alamosensis* (Coulter) Gibson & Horak*[Rathbunia alamosensis]*	Alamosogenin	AP	[109,110]
		Gummosogenin		
	*S. aragonii* (Weber) Buxbaum	β-amyrin	AP	[111]
	*S. benekei* (Ehrenberg) Buxbaum	Lupeol	AP	[112,113,114]
		Oleanolic acid		
		β-amyrin		
		Lupeone		
		Queretaroic acid		
	*S. eichlamii* (Britton & Rose) Buxbaum	Oleanolic acid	AP	[115]
		Erythrodiol		
		Longispinogenin		
	*S. euruca* (Brandegee) Gibson & Horak	Oleanolic acid	AP	[111,116,117,118]
		Betulinic acid		
		Stellatogenin		
		Turberogenin		
		Machaerogenin		
		Machaeric acid		
		21-ketobetulinic acid		
		16β-hydroxybetulinic acid		
		22β-hydroxistellatogenin		
		Morolic acid		
		Queretaroic acid		
		27-desoxyfillirigenin		
		Treleasegenic acid		
	*S. fimbriatus* (Lamark) Lourteig	Oleanolic acid	AP	[119]
		Betulinic acid		
		Erythrodiol		
		Longispinogenin		
	*S. griseus* (Haworth) Buxbaum	Oleanolic acid	AP	[98]
		Betulin		
		Betulinic acid		
		Erythrodiol		
		Longispinogenin		
	*S. gummosus* (Brandegee) Gibson & Horak	Gummosogenin	AP	[109,111,120]
		Machaeric acid		
		Macherinic acid		
	*S. pruinosus* (Otto) Buxbaum	Oleanolic acid	AP	[32,95,111]
		Erithrodiol		
		Longispinogenin		
		3β-hydroxi-11α,12α-epoxyolean-28,13β-olide		
	*S. queretaroensis* (Weber) Buxbaum	Oleanolic acid	AP	[114]
		Queretaroic acid		
	*S. quevedonis* (Ortega) Bubaum	Oleanolic acid	AP	[98]
		Betulinic acid		
		Longispinogenin		
	*S. stellatus* (Pfeiffer) Riccobono	Oleanolic acid	AP	[98,116,121]
		Betulinic acid		
		Stellatogenin		
		Turberogenin		
		Machaerogenin		
		Queretaroic acid		
		16β-hydroxistellatogenin		
	*S. thurberi* (Engelmann) Buxbaum	Lupeol	AP	[50,111,113,114,122,123,124,125]
		Oleanolic acid		
		Betulin		
		Maniladiol		
		Erithrodiol		
		Longispinogenin		
		β-amyrin		
		Oleanolic aldehyde		
		Turberogenin		
		Queretaroic acid		
		Calenduladiol		
		Betulinic aldehyde		
	*S. treleasei* (Britton & Rose) Backeberg	Oleanolic acid	AP	[111,126]
		Longispinogenin		
		Treleasegenic acid		

^1^ AP: aerial parts; R: roots; P: pollen.

**Table 9:** Most representative biological activities of triterpenes isolated from Mexican columnar Cactaceae.

Compound	Activity	Description	Reference
Lupeol	AD, AO, AN, AI, CT, RN, HP	AD = moderate inhibition of α-Glucosidase and α-Amylase and selective allosteric inhibition of PTP1B. AO = decreases ROS and LPO generation. AN = growth inhibitory activity against several bacteria as *M. smegmatis, M. aurum*, and *E. faecalis*, the parasites *P. falciparum* and *L. donovani*, and the virus HSV-1 and the reverse transcriptase of HIV-1. AI = inhibition of IL-1β and NF-κB produced an inhibitory effect on the carrageenan-induced edema assay. CT = inhibits cell growth by several mechanisms: inhibition of the phosphorylation of ECFR, Topoisomerase and WNT/β-catenin regulation, induction of cell cycle arrest and mitochondria-mediated apoptosis. IN = moderate larvicidal activity against A. aegypti. RN = strong protective effect of cisplatin-induced nephrotoxicity by upregulating the phosphorylation of MAPKs. HP = alleviate liver injury by GalN/LPS through suppression of the IRAK-mediated TLR4 signal pathway.	[13,87,127,128,129,130,131,132,133,134,135,136,137,138,139,140,141,142,143,144]
Oleanolic acid	AD, AN, AI, CR, CT, IM, HP	AD = strong regulation of PPARγ and miR-98-5p/PGC-1b axis causing a hypolipidemic effect. AN = growth inhibitory activity against several bacteria as S. aureus, M. smegmatis, E. faecalis, L. monocytogenes, B. cereus, and P. berghei and the parasite Leishmania spp. and inhibitory activity against the reverse transcriptase of HIV-1. AI = inhibition of Lipoxygenase and phospholipase A2 activity. CR = modulation of the BDNF-ERK1/2-CREB pathway through TrkB activation. CT = inhibition of cervical (HeLa), ovary (SK-OV-3), breast (MCF-7), colon (DLD-1) cancer cell lines proliferation. IM = reduce the synthesis of pro-inflammatory mediators, auto-antibody production, suppression of endogenous leptin production, and inhibits migration of leukocytes in the CNS. OT = increase bone mineral density. Its effect is associated with effects on Ca and vitamin D metabolism. HP = showed moderate activity towards in vitro immunological liver injury and low hepatotoxicity.	[13,136,145,146,147,148,149,150,151,152,153,154,155]
Betulin	AD, AN, CR, AI, CT	AD = selective allosteric inhibition of PTP1B, noncompetitive inhibitor of α-amylase and inhibition of α-glucosidase. AN = growth inhibitory activity against several bacteria as M. smegmatis and M. aurum, the parasite P. falciparum, and the virus HSV-1 and the reverse transcriptase of HIV-1. CR = protective effect on cognition inhibiting the NF-κB pathway, by regulation of the GABAA receptor, and by moderate cholinesterase inhibition. It also reduced 6-hydroxydopamine-induced dopaminergic neuron degeneration. AI = decreases NO production, iNOS expression in vitro, and NF-κB activity. It also decreases the levels of MPO, IL-1β, and TNF-α in liver tissue. CT = strong inhibitory effect on the proliferation of several cell lines triggering apoptosis by mitochondrial pathway and NOXA induction.	[87,127,133,137,138,139,143,156,157,158,159,160,161,162,163,164,165,166]
Betulinic acid	AD, AN, IN, AI, CT, RN, HP	AD = selective allosteric inhibition of PTP1B, noncompetitive inhibitor of α-amylase and inhibition of α-glucosidase. It also stimulates insulin secretion by the mediation of potassium and chloride channels. AN = growth inhibitory activity against several bacteria as M. smegmatis, M. aurum, S. aureus, B subtilis, E. faecalis, and B. cereus, the parasites P. falciparum and T. cruzi, and the virus HSV-1 and the reverse transcriptase inhibitor of HIV-1. IN = inhibitor of tyrosinase and could be used as an insecticidal agent. CR = neuroprotective effect on cognition by moderate cholinesterase inhibition. It also improves cAMP, cGMP and BDNF levels. AI = decreases NO production and iNOS and IL-6 expression in vitro. CT = inhibition of cervical (HeLa), ovary (SK-OV-3), breast (MCF-7), colon (DLD-1) cancer cell lines proliferation. It has also shown to induce apoptosis by DNA damage, G2/M cell cycle arrest, and Bcl-2/Bax signaling regulation. RN = strong protective effect of cisplatin-induced nephrotoxicity by upregulating the phosphorylation of MAPKs. HP = inhibition of liver oxidative stress in the iron/ascorbate system and showed hepatoprotective effects against D-GalN/TNF-α induced cell dead.	[13,19,87,127,133,135,136,137,138,143,147,160,161,163,167,168,169,170,171]
Maniladiol	AN, AI	AN = growth inhibitory activity against M. tuberculosis and the reverse transcriptase inhibition of HIV-1. AI = decreases the production of pro-inflammatory cytokines such as TNF-α, IL-1, and IL-6 and inhibits NO production.	[164,172,173]
Erythrodiol	AN, CT, HT, IM, HP	AN = growth inhibitory activity against several bacteria as B. subtilis, E. coli, and C. albicans and the reverse transcriptase inhibitor of HIV-1. Inhibitor of tyrosinase and could be used as an insecticidal agent AI = edema inhibition in the TPA-induced inflammation assay. CT = potent anti-proliferative effect inducing apoptosis, cell cycle arrest, and ROS generation. HT = reduce cardiac remodeling by inhibiting angiotensin II-induced proliferation via PPAR-γ. IM = reduce the synthesis of pro-inflammatory mediators, auto-antibody production, suppression of endogenous leptin production, and inhibits migration of leukocytes in the CNS. HP = high protection of human hepatoma cells against CCl4-induced injury with ALT level decreased.	[13,153,164,167,174,175,176,177,178,179]
Longispinogenin	AN, AI, CT	AN = growth inhibitory activity against M. tuberculosis. AI = edema inhibition in the TPA-induced inflammation assay. CT = inhibition of cervical (HeLa) cancer cell line proliferation.	[173,180,181]
Pachanol D	AC	AC = strong inhibitory effect on the acetic acid-induced writhing test.	[95]
Cochalic acid	CT	CT = potent inhibition of cervical (HeLa) cancer cell line proliferation.	[180]
Chichipegenin	AI, CT	AI = edema inhibition in the TPA-induced inflammation assay. CT = inhibition of breast and colon carcinoma MCF-7 and HCT-15 cell lines proliferation.	[26]
Stellatogenin	AC	AC = strong inhibitory effect on the acetic acid-induced writhing test.	[95]
β-amyrin	AD, AN, AI, CR, CT, HP	AD = moderate inhibition of α-Glucosidase and α-Amylase. AN = growth inhibitory activity against several bacteria as B. subtilis, S. aureus, and C. albicans, the parasite T. cruzi, and antiviral inhibitory activity against the reverse transcriptase of HIV-1 and IAV. AI = decreases the production of pro-inflammatory cytokines such as TNF-α, IL-1, and IL-6 and inhibits NO production CR = interaction with the GABAA receptor and produce sedative and hypnotic, increasing noradrenergic activity. It also showed neuroprotective activity reducing α-synuclein aggregation upregulating LGG-1 expression. CT = inhibition of proliferation in the cervical (HeLa), ovary (SK-OV-3), breast (MCF-7), colon (DLD-1), and other cancer cell lines. HP = hepatoprotective effect against acetaminophen-induced hepatotoxicity.	[143,147,164,171,172,182,183,184,185,186]
Oleanolic aldehyde	AD, AN	AD = dose-dependent enhancement of insulin secretion by INS-1 cells. AN = growth inhibitory activity against S. mutans and P. gingivalis.	[187,188]
Gummosogenin	AC, CT	AC = strong inhibitory effect on the acetic acid-induced writhing test. CT = inhibition of cervical (HeLa) cancer cell line proliferation.	[95,180]
Lupeone	AN, AD, AI, RN	AN = reverse transcriptase inhibitor of HIV-1. AD = moderate inhibition of α-Glucosidase and selective allosteric inhibition of PTP1B. AI = decreases the production of pro-inflammatory cytokines such as IL-12 and IL-6. RN = strong protective effect of cisplatin-induced nephrotoxicity.	[128,133,135,164,183,189]
Turberogenin	AC, CT	AC = strong inhibitory effect on the acetic acid-induced writhing test. CT = low inhibition of cervical (HeLa) cancer cell line proliferation.	[95,180]
Morolic acid	AD, AI	AD = induced a significant reduction of blood glucose levels by inhibition of 11β-HSD1. AI = inhibition of the leukocyte dermal infiltration and inhibition of key inflammatory enzymes as PLA2 and 5-LOX.	[190,191]
Queretaroic acid	CT	CT = inhibition of cervical (HeLa) cancer cell line proliferation.	[180]
Calenduladiol	CR, CT	CR = inhibition of acetylcholinesterase and butyrylcholinesterase in vitro. CT = inhibition of leukemia (NB4 and K562) cancer cell lines proliferation.	[192,193]
Cycloartenol	AD, AN, and CT	AD = Decrease glucose intestinal absorption that could be associated with SGLT1 regulation as well as α-glucosidase inhibition. Downregulation of fatty acid synthesis and interferes with the absorption of cholesterol. AN = Inhibitory effect against bacteria E. coli and P. aeruginosa and low inhibition of the parasite *P. falciparum*. CT = Weak cytotoxic activity against HL60 cell line and p38MAPK-mediated apoptosis in the U87 cell line.	[91,194,195,196,197,198,199,200]
Betulinic aldehyde	AN, CT	AN = growth inhibitory activity against bacteria *P. smartii, E. faecalis, S. aureus, and E. coli* and antiviral activity against Influenza virus (KBNP-0028) and the reverse transcriptase of HIV-1. CT = inhibition of cervical (HeLa), ovary (SK-OV-3), breast (MCF-7), colon (HCT-116) and melanoma (SK-MEL-5) cancer cell lines proliferation.	[88,135,164,201]

AD = Antidiabetic, AM = Antimutagenic, AO = Antioxidant, AN = Anti-infective, CR = CNS Regulation, AI = Anti-inflammatory, CT = Citotoxic, MM = Modulation of Cholesterol metabolism, HT = Hypertension, IN = insecticidal, IM = Immune modulation, RN = renoprotective, AC = Anti nociceptive, HP = Hepatoprotective.

**Table 10:** Top-10 ranked sterol compounds in the COX-1 and COX-2 docking study.

Ligand	COX-1	COX-2
Thurberol	−132.1	−144.1
Locereol	−133.1	−141.1
Fucosterol	−130.6	−141.7
5α-cholesta-8,14-dien-3 β-ol	−130.4	−141.5
Spinasterol	−130.6	−138.5
24-methylenecolesterol	−127.3	−139.9
β-sitosterol	−124.3	−136.9
Peniocerol	−124.3	−134.1
24-Methylenelophenol	−127.3	−130.7
Lophenol	−123.7	−131.2

**Table 11:** Top-10 ranked triterpene compounds in the COX-1 and COX-2 docking study.

Ligand	COX-1	COX-2
Lupeone	−104.0	−97.1
Thurberogenin	−92.3	−93.6
Lupeol	−97.7	−87.3
Betulinic aldehyde	−94.9	−88.6
16β-hydroxybetulinic acid	−82.9	−95.1
Calenduladiol	−92.8	−85.1
16β-hydroxystellatogenin	−93.8	−83.0
22β-hydroxystellatogenin	−94.0	−79.3
21-ketobetulinic acid	−81.6	−87.1
Machaerogenin	−87.0	−79.4

**Table 12:** Top-10 ranked sterol derivatives in the PTP1B, PPAR-α and PPAR-γ docking study.

LIGAND	PTP1B	PPAR-α	PPAR-γ
Fucosterol	−141.7	−135.7	−138.9
β-sitosterol	−131.6	−141.0	−142.4
Schottenol	−132.9	−139.0	−138.9
Spinasterol	−133.1	−140.8	−131.2
Thurberol	−135.4	−129.3	−140.4
Cyclostenol	−140.4	−137.3	−126.4
24-Methylenecholesterol	−132.7	−133.5	−136.0
Peniocerol	−132.4	−127.9	−140.5
Opuntisterol	−126.0	−141.5	−132.7
Steneocerol	−133.0	−136.2	−126.2

**Table 13:** Top-10 ranked triterpene derivatives in the PTP1B, PPAR-α and PPAR-γ docking study.

LIGAND	PTP1B	PPAR-α	PPAR-γ
16β-hydroxystellatogenin	−98.5	−128.7	−119.5
Thurberogenin	−104.8	−123.4	−114.8
Stellatogenin	−99.4	−124.8	−116.9
Myrtillogenic acid	−95.4	−123.8	−119.3
Alamosogenin	−98.1	−120.5	−118.2
22β-hydroxystellatogenin	−93.4	−130.0	−112.6
Oleanolic acid	−101.6	−118.9	−114.2
Machaeric acid	−105.2	−112.3	−115.6
Oleanolic aldehyde	−100.4	−116.8	−115.6
Machaerinic acid	−103.8	−114.0	−114.8

**Table 14:** Top-10 ranked sterol derivatives in the LXR-α, LXR-β and acetylcholinesterase (AChE) docking study.

LIGAND	LXR-α	LXR-β	AChE
Fucosterol	−167.1	−167.7	−152.9
β-sitosterol	−165.2	−164.3	−151.7
Methylenecolesterol	−163.1	−157.8	−148.6
Thurberol	−161.9	−158.7	−147.5
Spinasterol	−157.0	−158.2	−152.6
Opuntisterol	−155.9	−157.9	−152.1
Cyclostenol	−155.4	−158.2	−147.7
Peniocerol	−158.6	−155.8	−143.4
Schottenol	−155.9	−153.6	−147.4
24-Methylenelophenol	−155.7	−154.6	−146.3

**Table 15:** Top-10 ranked triterpene derivatives in the LXR-α, LXR-β and AChE docking study.

LIGAND	LXR-α	LXR-β	AChE
Thurberogenin	−153.915	−155.492	−143.2
16β-hydroxystellatogenin	−149.154	−152.308	−134.8
Betulinic acid	−141.254	−155.181	−137.3
Stellatogenin	−142.879	−150.498	−139.4
16β-hydroxybetulinic acid	−145.372	−156.064	−127.6
Calenduladiol	−147.818	−152.858	−127.8
Betulin	−141.428	−153.007	−133.0
Lupenone	−145.652	−146.987	−134.2
Lupeol	−146.667	−148.674	−129.8
Alamosogenin	−141.635	−144.049	−138.3

## Supplementary Materials

The following are available online, Table S1: Complete docking results from the in silico bioprospection on compounds isolated from Mexican Columnar Cactaceae.

## References

[1] Reyes-Santiago J. (2009). Conservación y restauración de cactáceas y otras plantas suculentas mexicanas. Manual Práctico. CONAFOR y SEMARNAT, México. https://www.conafor.gob.mx/biblioteca/Manual_Practico-Conservacionyrestauracion-cactaceas_suculentas.pdf.

[2] Bravo-Hollis H. (1978). Las Cactáceas de México.

[3] Anderson E.F. (2001). The Cactus Family.

[4] Guzmán U., Arias S., Dávila P. (2007). Catálogo de autoridades taxonómicas de las cactáceas (Cactaceae: Magnoliopsida) de México. http://www.snib.mx/descargasSNIBmx/SNIBTaxonomia_20200329_142957.zip.

[5] Batis A., Rojas M. (2002). El Peyote Y Otros Cactos Alucinógenos De Mexico. Biodoversitas.

[6] De Wit M., du Toit A., Osthoff G., Hugo A. (2019). Cactus pear antioxidants: A comparison between fruit pulp, fruit peel, fruit seeds and cladodes of eight different cactus pear cultivars (Opuntia ficus-indica and Opuntia robusta). J. Food Meas. Charact..

[7] Berrabah H., Taïbi K., Ait Abderrahim L., Boussaid M. (2019). Phytochemical composition and antioxidant properties of prickly pear (*Opuntia ficus-indica* L.) flowers from the Algerian germplasm. J. Food Meas. Charact..

[8] Harrat N.e.l., Louala S., Bensalah F., Affane F., Chekkal H., Lamri-Senhadji M. (2019). Anti-hypertensive, anti-diabetic, hypocholesterolemic and antioxidant properties of prickly pear nopalitos in type 2 diabetic rats fed a high-fat diet. Nutr. Food Sci..

[9] Aruwa C.E., Amoo S.O., Kudanga T. (2018). Opuntia (Cactaceae) plant compounds, biological activities and prospects—A comprehensive review. Food Res. Int..

[10] Aragona M., Lauriano E.R., Pergolizzi S., Faggio C. (2018). *Opuntia ficus* - *indica* (L.) Miller as a source of bioactivity compounds for health and nutrition. Nat. Prod. Res..

[11] Feugang J.M., Konarski P., Zou D., Stintzing F.C., Zou C. (2006). Nutritional and medicinal use of Cactus pear (Opuntia spp.) cladodes and fruits. Front. Biosci..

[12] Santos-Díaz M.S., Camarena-Rangel N.G. (2019). Cacti for production of metabolites: Current state and perspectives. Appl. Microbiol. Biotechnol..

[13] Akihisa T., Yasukawa K. (2001). Antitumor-promoting and anti-inflammatory activities of triterpenoids and sterols from plants and fungi. Stud. Nat. Prod. Chem..

[14] Harlev E., Nevo E., Solowey E., Bishayee A. (2013). Cancer preventive and curative attributes of plants of the Cactaceae family: A review. Planta Med..

[15] Kontoyianni M. (2017). Docking and Virtual Screening in Drug Discovery. Methods Mol. Biol..

[16] Yusuf M., Hardianto A., Muchtaridi M., Nuwarda R.F., Subroto T. (2019). Introduction of Docking-Based Virtual Screening Workflow Using Desktop Personal Computer. Encycl. Bioinforma. Comput. Biol..

[17] Loza-Mejía M.A., Salazar J.R. (2015). Sterols and triterpenoids as potential anti-inflammatories: Molecular docking studies for binding to some enzymes involved in inflammatory pathways. J. Mol. Graph. Model..

[18] Loza-Mejía M., Salazar J., Sánchez-Tejeda J. (2018). In Silico Studies on Compounds Derived from Calceolaria: Phenylethanoid Glycosides as Potential Multitarget Inhibitors for the Development of Pesticides. Biomolecules.

[19] Thao N.P., Kim J.H., Thuy Luyen B.T., Dat N.T., Kim Y.H. (2017). In silico investigation of cycloartane triterpene derivatives from Cimicifuga dahurica (Turcz.) Maxim. roots for the development of potent soluble epoxide hydrolase inhibitors. Int. J. Biol. Macromol..

[20] Ochoa R., García E., Robledo S.M., Cardona G W. (2019). Virtual and experimental screening of phenylfuranchalcones as potential anti-Leishmania candidates. J. Mol. Graph. Model..

[21] Wang Y., Sun Y., Wang Y., Ju Y., Meng D. (2019). Virtual screening of active compounds from Artemisia argyi and potential targets against gastric ulcer based on Network pharmacology. Bioorg. Chem..

[22] Dawood H.M., Ibrahim R.S., Shawky E., Hammoda H.M., Metwally A.M. (2018). Integrated in silico-in vitro strategy for screening of some traditional Egyptian plants for human aromatase inhibitors. J. Ethnopharmacol..

[23] Huang H., Chu C.-L., Chen L., Shui D. (2019). Evaluation of potential inhibitors of squalene synthase based on virtual screening and in vitro studies. Comput. Biol. Chem..

[24] Medina-Franco J.L. (2019). New Approaches for the Discovery of Pharmacologically-Active Natural Compounds. Biomolecules.

[25] Prieto-Martínez F., Medina-Franco J., Prieto-Martínez F.D., Medina-Franco J.L. (2018). Flavonoids as Putative Epi-Modulators: Insight into Their Binding Mode with BRD4 Bromodomains Using Molecular Docking and Dynamics. Biomolecules.

[26] Salazar J.R., Martinez-Vazquez M., Ramirez-Apan T., Nieto-Camacho A., Cespedes C.L., Rodrfguez-Silverio J., Flores-Murrieta F. (2011). Anti-Inflammatory and Cytotoxic Activities of Chichipegenin, Peniocerol, and Macdougallin Isolated from Myrtillocactus geometrizans (Mart. ex Pfeiff.) Con. Z. Fur Nat. Sect. C J. Biosci..

[27] Salazar J.R., Céspedes C.L. (2013). Phytoecdysteroids and related sterols isolated from mexican cacti: Their potential use as natural insecticides. Natural Antioxidants and Biocides from Wild Medicinal Plants.

[28] Torres-Olvera M., Salazar J.R., Soto-Cabrera D., Cerón-Nava A., Rosales-Guevara J. (2014). Evaluation of the antimicrobial activity of extracts and compounds isolated from *Hylocereus* sp.. Vitae.

[29] Uribe-Chiquete R.F., Salazar J.R., Ariza-Castolo A., Ramos-Gonzales V.H. (2014). Antimicrobial activity of methanolic extract, peniocerol and longispinogenin extracted from Myrtillocactus geometrizans. Vitae.

[30] Nogueda-Gutiérrez I.B., Salazar J.R., Cerón-Nava A., Ramírez-Ponce A.L., Torres-Olvera M., Soto-Cabrera D., Ciprés-Meixueiro A. (2014). Quantification of flavonoids and antioxidant and antimicrobial activities of the extract of Peniocereus maculatus. Vitae.

[31] Ramírez-Ponce A.L., Salazar J.R., Cerón-Nava A., Torres-Olvera M., Soto-Cabrera D., Nogueda-Gutiérrez I.B. (2014). Quantification of total polyphenols, flavonoids and evaluation of the antioxidant and antimicrobial activities of Opuntia tomentosa extract. Vitae.

[32] Soto-Cabrera D., Salazar J.R., Nogueda-Gutiérrez I., Torres-Olvera M., Cerón-Nava A., Rosales-Guevara J., Terrazas T., Rosas-Acevedo H. (2016). Quantification of polyphenols and flavonoid content and evaluation of anti-inflammatory and antimicrobial activities of Stenocereus stellatus extracts. Nat. Prod. Res..

[33] Céspedes C.L., Salazar J.R., Martínez M., Aranda E. (2005). Insect growth regulatory effects of some extracts and sterols from Myrtillocactus geometrizans (Cactaceae) against Spodoptera frugiperda and Tenebrio molitor. Phytochemistry.

[34] Pattee A.F. (1867). Cereus Grandiflora, Cactus Grandiflora (Linn.), Night-Blooming cereus, Sweet-Scented Cactus, &c. Bost. Med. Surg. J..

[35] Britton N.L., Rose J.N. (1919). The Cactaceae: Descriptions and Illustrations of Plants of the Cactus Family.

[36] Heyl G. (1901). Ueber das Vorkommen von Alkaloiden und Saponinen in Cacteen. Arch. Pharm. (Weinh.).

[37] Ewell E.E. (1896). THE CHEMISTRY OF THE CACTACEAE. J. Am. Chem. Soc..

[38] Nyffeler R., Eggli U. (2010). A farewell to dated ideas and concepts: Molecular phylogenetics and a revised suprageneric classification of the family Cactaceae - Zurich Open Repository and Archive. Schumannia.

[39] Fogleman J.C., Duperret S.M., Kircher H.W. (1986). The role of phytosterols in host plant utilization by cactophilicDrosophila. Lipids.

[40] Fogleman J.C., Armstrong L. (1989). Ecological aspects of cactus triterpene glycosides I. Their effect on fitness components ofDrosophila mojavensis. J. Chem. Ecol..

[41] Fogleman J.C., Danielson P.B. (2001). Chemical Interactions in the Cactus-Microorganism-Drosophila Model System of the Sonoran Desert. American Zoologist.

[42] Djerassi C., Krakower G.W., Lemin A.J., Liu L.H., Mills J.S., Villotti R. (1958). The Neutral Constituents of the Cactus Lophocereus schottii. The Structure of Lophenol 4α-Methyl-Δ7-cholesten-3β-ol, A Link in Sterol Biogenesis. J. Am. Chem. Soc..

[43] Campbell C.E., Kircher H.W. (1980). Senita cactus: A plant with interrupted sterol biosynthetic pathways. Phytochemistry.

[44] Djerassi C., Knight J.C., Brockmann H. (1964). Neue Sterine aus dem KaktusWilcoxia viperina. Chem. Ber..

[45] Knight J.C., Wilkinson D.I., Djerassi C. (1966). The Structure of the Cactus Sterol Macdougallin (14α-Methyl-Δ^8^ -cholestene-3β,6α-diol). A Novel Link in Sterol Biogenesis ^1,2^. J. Am. Chem. Soc..

[46] Arias S., Terrazas T., Arreola-Nava H.J., Vázquez-Sánchez M., Cameron K.M. (2005). Phylogenetic relationships in Peniocereus (Cactaceae) inferred from plastid DNA sequence data. J. Plant. Res..

[47] Djerassi C., Murray R.D.H., Villotti R. (1961). The Structure of the Cactus Sterol, Peniocerol (Cholest-8ene-3~,6a-diol). Proc. Chem. Soc..

[48] Djerassi C., Knight J.C., Brockmann H. (1963). The Structure of the Cactus Sterol Macdougallin (14α-Methyl-Δ^8^-Cholestene-3β,6α-Diol).A Novel Link in Sterol Biogenesis. J. Am. Chem. Soc..

[49] Knight J.C., Pettit G.R. (1969). Arizona flora: The sterols of Peniocereus greggii. Phytochemistry.

[50] Kircher H.W. (1980). Triterpenes in organ pipe cactus. Phytochemistry.

[51] Kircher H.W., Bird H.L. (1982). Five 3β, 6α-dihydroxysterols in organ-pipe cactus. Phytochemistry.

[52] Jiang J., Li Y., Chen Z., Min Z., Lou F. (2006). Two novel C29-5beta-sterols from the stems of Opuntia dillenii. Steroids.

[53] Dinan L. (2001). Phytoecdysteroids: Biological aspects. Phytochemistry.

[54] Dinan L., Savchenko T., Whiting P. (2001). On the distribution of phytoecdysteroids in plants. Cell. Mol. Life Sci..

[55] Salt T.A., Tocker J.E., Adler J.H. (1987). Dominance of Δ5-sterols in eight species of the cactaceae. Phytochemistry.

[56] Schaller H. (2003). The role of sterols in plant growth and development. Prog. Lipid Res..

[57] Jayasuriya H., Herath K.B., Ondeyka J.G., Guan Z., Borris R.P., Tiwari S., De Jong W., Chavez F., Moss J., Stevenson D.W. (2005). Diterpenoid, steroid, and triterpenoid agonists of liver X receptors from diversified terrestrial plants and marine sources. J. Nat. Prod..

[58] Lusby W.R., Buchmann S.L., Feldlaufer M.F. (1993). Pollen sterols from three species of sonoran cacti. Lipids.

[59] Standifer L.N., Devys M., Barbier M. (1968). Pollen sterols—A mass spectrographic survey. Phytochemistry.

[60] Nes W.D., Schmidt J.O. (1988). Isolation of 25(27)-dehydrolanost-8-enol from Cereus giganteus and its biosynthetic implications. Phytochemistry.

[61] Nomaguchi K., Tanaka M., Misawa E., Yamada M., Toida T., Iwatsuki K., Goto T., Kawada T. (2011). Aloe vera phytosterols act as ligands for PPAR and improve the expression levels of PPAR target genes in the livers of mice with diet-induced obesity. Obes. Res. Clin. Pr..

[62] Misawa E., Tanaka M., Nomaguchi K., Nabeshima K., Yamada M., Toida T., Iwatsuki K. (2012). Oral Ingestion of Aloe vera Phytosterols Alters Hepatic Gene. J. Agric. Food Chem..

[63] Gomez-Flores R., Quintanilla-Licea R., Hernández-Martínez H.C., Samaniego-Escamilla M., Tamez-Guerra P., Monreal-Cuevas E., Tamez-Guerra R., Rodriguez-Padilla C. (2019). Survival of Lymphoma-Bearing Mice by Pachycereus marginatus Cactus Extracts and Elucidation of Bioactive Compounds. Nat. Prod. Commun..

[64] El Kharrassi Y., Samadi M., Lopez T., Nury T., El Kebbaj R., Andreoletti P., El Hajj H.I., Vamecq J., Moustaid K., Latruffe N. (2014). Biological activities of Schottenol and Spinasterol, two natural phytosterols present in argan oil and in cactus pear seed oil, on murine miroglial BV2 cells. Biochem. Biophys. Res. Commun..

[65] Han Y.H., Ham J.H., Lee N.J., Park C.H., Shin Y.H., Lee D.U. (2000). Antimutagenic activity of 5α-cholest-7-en-3β-ol, a new component from the starfish Asterina pectinifera. Biol. Pharm. Bull..

[66] Raslan A.E., Radwan M.M., Ahmed S.A., Nafady A.M., Zaki M.A., Wanas A.S., Abou-Karam M., Shier T.W., Hassanean H.A., ElSohly M.A. (2018). Monanchoramides A–D, ceramides from the marine sponge Monanchora clathrata with cytotoxic activity. Phytochem. Lett..

[67] Socała K., Wlaź P. (2016). Evaluation of the antidepressant- and anxiolytic-like activity of α-spinasterol, a plant derivative with TRPV1 antagonistic effects, in mice. Behav. Brain Res..

[68] Socała K., Nieoczym D., Pieróg M., Wlaź P. (2015). α-Spinasterol, a TRPV1 receptor antagonist, elevates the seizure threshold in three acute seizure tests in mice. J. Neural Transm..

[69] Meneses-Sagrero S.E., Navarro-Navarro M., Ruiz-Bustos E., Del-Toro-Sánchez C.L., Jiménez-Estrada M., Robles-Zepeda R.E. (2017). Antiproliferative activity of spinasterol isolated of Stegnosperma halimifolium (Benth, 1844). Saudi Pharm. J..

[70] Brusco I., Camponogara C., Carvalho F.B., Schetinger M.R.C., Oliveira M.S., Trevisan G., Ferreira J., Oliveira S.M. (2017). α-Spinasterol: A COX inhibitor and a transient receptor potential vanilloid 1 antagonist presents an antinociceptive effect in clinically relevant models of pain in mice. Br. J. Pharm..

[71] Wang Y.C., Li W.Y., Wu D.C., Wang J.J., Wu C.H., Liao J.J., Lin C.K. (2011). In vitro activity of 2-methoxy-1,4-naphthoquinone and stigmasta-7,22-diene- 3β-ol from *Impatiens balsamina* L. against multiple antibiotic-resistant Helicobacter pylori. Evid. Based Complement. Altern. Med..

[72] Egas V., Salazar-Cervantes G., Romero I., Méndez-Cuesta C.A., Rodríguez-Chávez J.L., Delgado G. (2018). Anti-Helicobacter pylori metabolites from Heterotheca inuloides (Mexican arnica). Fitoterapia.

[73] Sedky N.K., El Gammal Z.H., Wahba A.E., Mosad E., Waly Z.Y., El-Fallal A.A., Arafa R.K., El-Badri N. (2018). The molecular basis of cytotoxicity of α-spinasterol from Ganoderma resinaceum: Induction of apoptosis and overexpression of p53 in breast and ovarian cancer cell lines. J. Cell. Biochem..

[74] Bolaños-Carrillo M.A., Ventura-Gallegos J.L., Saldivar-Jiménez A.D., Zentella-Dehesa A., Martínez-Vázquez M. (2015). Effect of Sterols Isolated from Myrtillocactus geometrizans on Growth Inhibition of Colon and Breast Cancer Cells. Evid. Based Complement. Altern. Med..

[75] Via L., Garcia-Argaeza A., Martínez-Vázquez M., Grancara S., Martinis P., Toninello A. (2014). Mitochondrial Permeability Transition as Target of Anticancer Drugs. Curr. Pharm. Des..

[76] Lajter I., Pan S.P., Nikles S., Ortmann S., Vasas A., Csupor-Löffler B., Forgó P., Hohmann J., Bauer R. (2015). Inhibition of COX-2 and NF- κ B1 Gene Expression, NO Production, 5-LOX, and COX-1 and COX-2 Enzymes by Extracts and Constituents of Onopordum acanthium. Planta Med..

[77] Li Y.-H., Yang Y.-F., Li K., Jin L.-L., Yang N.-Y., Kong D.-Y. (2009). 5 Alpha-Reductase and Aromatase Inhibitory Constituents from Brassica rapa L. Pollen. Chem. Pharm. Bull. (Tokyo).

[78] Viegelmann C., Parker J., Ooi T., Clements C., Abbott G., Young L., Kennedy J., Dobson A.D.W., Edrada-Ebel R.A. (2014). Isolation and identification of antitrypanosomal and antimycobacterial active steroids from the sponge Haliclona simulans. Mar. Drugs.

[79] Zhen X.H., Quan Y.C., Jiang H.Y., Wen Z.S., Qu Y.L., Guan L.P. (2015). Fucosterol, a sterol extracted from Sargassum fusiforme, shows antidepressant and anticonvulsant effects. Eur. J. Pharm..

[80] Abdul Q.A., Choi R.J., Jung H.A., Choi J.S. (2016). Health benefit of fucosterol from marine algae: A review. J. Sci. Food Agric..

[81] Jung H.A., Bhakta H.K., Min B.S., Choi J.S. (2016). Fucosterol activates the insulin signaling pathway in insulin resistant HepG2 cells via inhibiting PTP1B. Arch. Pharm. Res..

[82] Lee M.A., Tan L., Yang H., Im Y.-G., Im Y.J. (2017). Structures of PPARγ complexed with lobeglitazone and pioglitazone reveal key determinants for the recognition of antidiabetic drugs. Sci. Rep..

[83] Jiang H., Li J., Chen A., Li Y., Xia M., Guo P., Yao S., Chen S. (2018). Fucosterol exhibits selective antitumor anticancer activity against hela human cervical cell line by inducing mitochondrial mediated apoptosis, cell cycle migration inhibition and downregulation of m-TOR/PI3K/Akt signalling pathway. Oncol. Lett..

[84] Perumal P., Sowmiya R., Prasanna kumar S., Ravikumar S., Deepak P., Balasubramani G. (2018). Isolation, structural elucidation and antiplasmodial activity of fucosterol compound from brown seaweed, *Sargassum linearifolium* against malarial parasite *Plasmodium falciparum*. Nat. Prod. Res..

[85] Wong C.H., Gan S.Y., Tan S.C., Gany S.A., Ying T., Gray A.I., Igoli J., Wan E., Pang S.M., Gany S.A. (2018). Fucosterol inhibits the cholinesterase activities and reduces the release of pro-inflammatory mediators in lipopolysaccharide and amyloid-induced microglial cells. J. Appl. Phycol..

[86] Le C.F., Kailaivasan T.H., Chow S.C., Abdullah Z., Ling S.K., Fang C.M. (2017). Phytosterols isolated from *Clinacanthus nutans* induce immunosuppressive activity in murine cells. Int. Immunopharmacol..

[87] Banzouzi J.T., Soh P.N., Ramos S., Hemez J., Soh P.N., Ramos S., Toto P., Hemez J. (2015). Samvisterin, a new natural antiplasmodial betulin derivative From Uapaca paludosa (Euphorbiaceae). J. Ethnopharmacol..

[88] Li J.-L., Lunga P.-K., Zhao Y.-L., Qin X.-J., Yang X.-W., Liu Y.-P., Luo X.-D. (2015). Antibacterial constituents from Melodinus suaveolens. Chin. J. Nat. Med..

[89] Rajavel T., Packiyaraj P., Suryanarayanan V., Singh S.K., Ruckmani K., Pandima Devi K. (2018). β-Sitosterol targets Trx/Trx1 reductase to induce apoptosis in A549 cells via ROS mediated mitochondrial dysregulation and p53 activation. Sci. Rep..

[90] GUPTA R., SHARMA A.K., DOBHAL M.P., SHARMA M.C., GUPTA R.S. (2011). Antidiabetic and antioxidant potential of β-sitosterol in streptozotocin-induced experimental hyperglycemia. J. Diabetes.

[91] Chithambo B., Noundou X.S., Krause R.W.M. (2017). Anti-malarial synergy of secondary metabolites from Morinda lucida Benth. J. Ethnopharmacol..

[92] Chen J., Jiao R., Jiang Y., Bi Y., Chen Z.-Y. (2014). Algal Sterols are as Effective as β-Sitosterol in Reducing Plasma Cholesterol Concentration. J. Agric. Food Chem..

[93] Loizou S., Lekakis I., Chrousos G.P., Moutsatsou P. (2010). β-Sitosterol exhibits anti-inflammatory activity in human aortic endothelial cells. Mol. Nutr. Food Res..

[94] Gibson A.C., Horak K.E. (1978). Systematic Anatomy and Phylogeny of Mexican Columnar Cacti. Ann. Mo. Bot. Gard..

[95] Kinoshita K., Akiba M., Saitoh M., Ye Y., Koyama K., Takahashi K., Kondo N., Yuasa H. (1998). Antinociceptive effect of triterpenes from cacti. Pharm. Biol..

[96] Takizawa T., Kenoshita K., Koyama K., Takahashi K., Kondo N., Yuasa H., Kawai K.I. (1993). A new type of triterpene from trichocereus pachanoi. J. Nat. Prod..

[97] Kinoshita K., Takizawa T., Koyama K., Takahashi K., Kondo N., Yuasa H., Kawai K.-I. (1995). New Triterpenes from Trichocereus pachanoi. J. Nat. Prod..

[98] Djerassi C., Bowers A., Burstein S., Estrada H., Grossman J., Herrán J., Lemin A.J., Manjarrez A., Pakrashi S.C. (1956). Terpenoids. XXII. 1 Triterpenes from Some Mexican and South American Plants2. J. Am. Chem. Soc..

[99] Djerassi C., Farkas E., Lemin A.J., Collins J.C., Walls F. (1954). Terpenoids. VI. Dumortierigenin, a New Triterpene Lactone from the Cactus Lemaireocereus dumortieri. J. Am. Chem. Soc..

[100] Djerassi C., Robinson C.H., Thomas D.B. (1956). Terpenoids. XXV. 1 The Structure of the Cactus Triterpene Dumortierigenin 2,3. J. Am. Chem. Soc..

[101] Kinoshita K., Koyama K., Takahashi K., Kondo N., Yuasa H. (2000). A new triterpenoid saponin from Isolatocereus dumortieri. J. Nat. Prod..

[102] Djerassi C., Burstein S., Estrada H., Lemin A.J., Lippman A.E., Manjarrez A., Monsimer H.G. (1957). Terpenoids. XXVIII. 1 The Triterpene Composition of the Genus Myrtillocactus2. J. Am. Chem. Soc..

[103] Sandoval A., Manjarrez A., Leeming P.R., Thomas G.H., Djerassi C. (1957). Terpenoids. XXX. The Structure of the Cactus Triterpene Chichipegenin. J. Am. Chem. Soc..

[104] Djerassi C., Monsimer H.G. (1957). Terpenoids. XXVII. 1 The Structure of the Cactus Triterpene Myrtillogenic Acid2. J. Am. Chem. Soc..

[105] Djerassi C., Thomas G.H., Monsimer H. (1955). Terpenoids. XVI. 1 The Constitution of the Cactus Triterpene Cochalic Acid. Partial Reductions of Methyl Diketoechinocystate2. J. Am. Chem. Soc..

[106] Djerassi C., Thomas G.H. (1954). Terpenoids. XII. Constitution of the cactus triterpene cochalic acid. Chem. Ind..

[107] Shamma M., Rosenstock P. (1959). The Triterpenes of Heliabravoa chende. J. Org. Chem..

[108] Khong P.W., Lewis K.G. (1975). New triterpenoid extractives from Lemaireocereus chichipe. Aust. J. Chem..

[109] Djerassi C., Geller L.E., Lemin A.J. (1954). Terpenoids. VIII ^1^. The Structures of the Cactus Triterpenes Gummosogenin and longispinogenin. J. Am. Chem. Soc..

[110] Takizawa T., Kinoshita K., Koyama K., Takahashi K., Kondo N., Yuasa H. (1995). A NEW TRITERPENE FROM RATHBUNIA ALAMOSENSZS skeleton named pachanane), from isolation of a new triterpene, alamoseno- glycosides of the aerial parts of Rathbunia on the molecular ion at mlz 472 EM ]’ ring C of 1. The ’, C-nrnr values of the the methyls. J. Nat. Prod..

[111] Djerassi C., Liu L.H., Farkas E., Lippman A.E., Lemin A.J., Geller L.E., McDonald R.N., Taylor B.J. (1955). Terpenoids. XI.1 investigation of nine cactus species. Isolation of two new triterpenes, stellatogenin and machaeric acid2. J. Am. Chem. Soc..

[112] Wollenweber E., Dörr M. (1995). Wax composition of the two cacti Hylocereus purpusii and Stenocereus beneckii. Biochem. Syst. Ecol..

[113] Djerassi C., Geller L.E., Lemin A.J. (1953). Terpenoids. I. The Triterpenes of the Cactus. J. Am. Chem. Soc..

[114] Djerassi C., Henry J.A., Lemin A.J., Rios T., Thomas G.H. (1956). Terpenoids. XXIV. The structure of the Cactustriterpene Queretaroic Acid. J. Am. Chem. Soc..

[115] Djerassi C., McDonald R.M., Lemin A.J. (1953). Terpenoids. III. The Isolation of Erythrodiol, Oleanolic Acid and a New Triterpene Triol. Loneispinoeenin, from the Cactus Lemaireocereus lonsispinus. J. Am. Chem. Soc..

[116] Koyama K., Yama T., Kinoshita K., Takahashi K., Kondo N., Yuasa H. (1993). New triterpenes from cactaceous plants. J. Nat. Prod..

[117] Okazaki S., Kinoshita K., Koyama K., Takahashi K., Yuasa H. (2007). New triterpene saponins from Stenocereus eruca (Cactaceae). J. Nat. Med..

[118] Ye Y., Kinoshita K., Koyama K., Takahashi K., Kondo N., Yuasa H. (1998). New triterpenes from Machaerocereus eruca. J. Nat. Prod..

[119] Djerassi C., Lippman A.E. (1954). Terpenoids. X. The Triterpenes of the Cactus Lemaireocereus hystrix. J. Am. Chem. Soc..

[120] Djerassi C.D., Lippman A.E. (1955). Terpenoids. XIII.1 the structures of the cactus triterpenes machaeric acid and machaerinic acid. J. Am. Chem. Soc..

[121] Imai T., Okazaki S., Kinoshita K., Koyama K., Takahashi K., Yuasa H. (2006). Triterpenoid saponins from cultural plants of Stenocereus stellatus (Cactaceae). J. Nat. Med..

[122] Marx M., Leclercq J., Tursch B., Djerassi C. (1967). Terpenoids. LX. Revised structures of the cactus triterpene lactones stellatogenin and thurberogenin. J. Org. Chem..

[123] Jolad S., Steelink C. (1969). Thurberin, a new pentacyclic triterpene from organ-pipe cactus. J. Org. Chem..

[124] Kaspryzyk Z., Pyrek J., Jolad S.D., Steelink C. (1970). The identity of calenduladiol and thurberin: A lupenediol found in marigold flowers and organ pipe cactus. Phytochemistry.

[125] Kircher H.W. (1977). Triterpene glycosides and queretaroic acid in organ pipe cactus. Phytochemistry.

[126] Djerassi C., Mills J.S. (1958). Terpenoids. XXXII. The Structure of the Cactus Triterpene Treleasegenic Acid. Ring Conformational Alterations in a Pentacyclic Triterpene. J. Am. Chem. Soc..

[127] Fomogne-fodjo M.C.Y., Ndinteh D.T., Olivier D.K., Kempgens P., Vuuren S. (2017). Van Secondary metabolites from Tetracera potatoria stem bark with anti- mycobacterial activity. J. Ethnopharmacol..

[128] Seong S.H., Roy A., Jung H.A., Jung H.J., Choi J.S. (2016). Protein tyrosine phosphatase 1B and α-glucosidase inhibitory activities of Pueraria lobata root and its constituents. J. Ethnopharmacol..

[129] Kakarla L., Katragadda S.B., Tiwari A.K., Kotamraju K.S., Madhusudana K., Kumar D.A., Botlagunta M. (2016). Free radical scavenging, α-glucosidase inhibitory and anti-inflammatory constituents from Indian sedges, *Cyperus scariosus* R.Br and *Cyperus rotundus* L.. Pharm. Mag..

[130] Moridi Farimani M., Nazarianpoor E., Rustaie A., Akhbari M. (2017). Phytochemical constituents and biological activities of Cleome iberica DC. Nat. Prod. Res..

[131] Srivastava A.K., Mishra S., Ali W., Shukla Y. (2016). Protective effects of lupeol against mancozeb-induced genotoxicity in cultured human lymphocytes. Phytomedicine.

[132] Rauth S., Ray S., Bhattacharyya S., Mehrotra D.G., Alam N., Mondal G., Nath P., Roy A., Biswas J., Murmu N. (2016). Lupeol evokes anticancer effects in oral squamous cell carcinoma by inhibiting oncogenic EGFR pathway. Mol. Cell. Biochem..

[133] Jin T., Yu H., Huang X.-F. (2016). Selective binding modes and allosteric inhibitory effects of lupane triterpenes on protein tyrosine phosphatase 1B. Sci. Rep..

[134] Gajos-Michniewicz A., Czyz M. (2016). Modulation of WNT/β-catenin pathway in melanoma by biologically active components derived from plants. Fitoterapia.

[135] Lee S., Jung K., Lee D., Rak S., Ro K., Sung K., Hyun K. (2015). Protective effect and mechanism of action of lupane triterpenes from Cornus walteri in cisplatin-induced nephrotoxicity. Bioorg. Med. Chem. Lett..

[136] Wang C.M., Chen H.T., Wu Z.Y., Jhan Y.L., Shyu C.L., Chou C.H. (2016). Antibacterial and synergistic activity of pentacyclic triterpenoids isolated from Alstonia scholaris. Molecules.

[137] Do Carmo D.F.M., Amaral A.C.F., Machado M., Lopes D., Echevarria A., Rosário V.E., Silva J.R.d.A. (2015). Evaluation of Antiplasmodial activity of extracts and constituents from Ampelozizyphus amazonicus. Pharm. Mag..

[138] Navid M.H., Laszczyk-lauer M.N., Reichling J., Schnitzler P. (2014). Phytomedicine Pentacyclic triterpenes in birch bark extract inhibit early step of herpes simplex virus type 1 replication. Eur. J. Integr. Med..

[139] Xu G.-m., Zan T., Li H.-y., Han J.-f., Liu Z.-m., Huang J., Dong L.-h., Zhang H.-n. (2018). Betulin inhibits lipopolysaccharide/D-galactosamine-induced acute liver injury in mice through activating PPAR-γ. Biomed. Pharm..

[140] Nobsathian S., Bullangpoti V., Kumrungsee N., Wongsa N., Ruttanakum D. (2018). Larvicidal effect of compounds isolated from Maerua siamensis (Capparidaceae) against Aedes aegypti (Diptera: Culicidae) larvae. Chem. Biol. Technol. Agric..

[141] Prasad N., Sabarwal A., Yadav U.C.S., Singh R.P. (2018). Lupeol induces S-phase arrest and mitochondria-mediated apoptosis in cervical cancer cells. J. Biosci..

[142] He W., Li X., Xia S. (2018). Lupeol triterpene exhibits potent antitumor effects in A427 human lung carcinoma cells via mitochondrial mediated apoptosis, ROS generation, loss of mitochondrial membrane potential and downregulation of m-TOR/PI3Ksol;AKT signalling pathway. J. Buon..

[143] Somtimuang C., Olatunji O.J., Ovatlarnporn C. (2018). Evaluation of In Vitro α-Amylase and α-Glucosidase Inhibitory Potentials of 14 Medicinal Plants Constituted in Thai Folk Antidiabetic Formularies. Chem. Biodivers..

[144] Kaur G., Chauhan K., Kaur S. (2019). Lupeol induces immunity and protective efficacy in a murine model against visceral leishmaniasis. Parasitology.

[145] Zhang Z., Jiang M., Xie X., Yang H., Wang X., Xiao L., Wang N. (2017). Oleanolic acid ameliorates high glucose-induced endothelial dysfunction via PPARδ activation. Sci. Rep..

[146] Nguyen H.T., Ho D.V., Vo H.Q., Le A.T., Nguyen H.M., Kodama T., Ito T., Morita H., Raal A. (2017). Antibacterial activities of chemical constituents from the aerial parts of Hedyotis pilulifera. Pharm. Biol..

[147] Mishra T., Arya R.K., Meena S., Joshi P., Pal M., Meena B., Upreti D.K., Rana T.S., Datta D. (2016). Isolation, characterization and anticancer potential of cytotoxic triterpenes from Betula utilis bark. PLoS ONE.

[148] Sibiya H.P., Mabandla M.V., Musabayane C.T. (2016). The effects of transdermally delivered oleanolic acid on malaria parasites and blood glucose homeostasis in P. berghei-infected male Sprague-Dawley rats. PLoS ONE.

[149] Chen S., Wen X., Zhang W., Wang C., Liu J., Liu C. (2017). Hypolipidemic effect of oleanolic acid is mediated by the miR-98-5p/PGC-1β axis in high-fat diet-induced hyperlipidemic mice. FASEB J..

[150] Jeon S.J., Lee H.J., Lee H.E., Park S.J., Gwon Y., Kim H., Zhang J., Shin C.Y., Kim D.H., Ryu J.H. (2017). Oleanolic acid ameliorates cognitive dysfunction caused by cholinergic blockade via TrkB-dependent BDNF signaling. Neuropharmacology.

[151] Giner-Larza E.M., Máez S., Recio M.C., Giner R.M., Prieto J.M., Cerdá-Nicolás M., Ríos J.L. (2001). Oleanonic acid, a 3-oxotriterpene from Pistacia, inhibits leukotriene synthesis and has anti-inflammatory activity. Eur. J. Pharm..

[152] Cao S., Dong X.L., Ho M.X., Yu W.X., Wong K.C., Yao X.S., Wong M.S. (2018). Oleanolic acid exerts osteoprotective effects and modulates vitamin D metabolism. Nutrients.

[153] Xu G., Xiao Y., Zhang Q., Zhou M. (2018). Hepatoprotective natural triterpenoids. Eur. J. Med. Chem..

[154] Montesino N.L., Schmidt T.J. (2018). Salvia species as sources of natural products with antiprotozoal activity. Int. J. Mol. Sci..

[155] Iranshahy M., Iranshahi M., Abtahi S.R., Karimi G. (2018). The role of nuclear factor erythroid 2-related factor 2 in hepatoprotective activity of natural products: A review. Food Chem. Toxicol..

[156] Chunhua M., Hongyan L. (2016). NeuroToxicology Protective effect of betulin on cognitive decline in streptozotocin (STZ) -induced diabetic rats. Neurotoxicology.

[157] Muceniece R., Saleniece K., Rumaks J., Krigere L., Dzirkale Z., Mezhapuke R., Zharkova O., Klusa V. (2008). Betulin binds to γ-aminobutyric acid receptors and exerts anticonvulsant action in mice. Pharm. Biochem. Behav..

[158] Król S.K., Kielbus M., Rivero-Müller A., Stepulak A. (2015). Comprehensive review on betulin as a potent anticancer agent. Biomed. Res. Int..

[159] Verma V., Tripathi A.C., Saraf S.K. (2016). Bioactive non-sterol triterpenoid from Streblus asper: Microwave-assisted extraction, HPTLC profiling, computational studies and neuro-pharmacological evaluation in BALB/c mice. Pharm. Biol..

[160] Laavola M., Haavikko R., Hämäläinen M., Leppänen T., Nieminen R., Alakurtti S., Moreira V.M., Yli-Kauhaluoma J., Moilanen E. (2016). Betulin Derivatives Effectively Suppress Inflammation in Vitro and in Vivo. J. Nat. Prod..

[161] Ratna Wulan D., Priyo Utomo E., Mahdi C. (2015). Antidiabetic Activity of Ruellia tuberosa L., Role of α-Amylase Inhibitor: In Silico, in Vitro, and in Vivo Approaches. Biochem. Res. Int..

[162] Zhang S.-Y., Zhao Q.-F., Fang N.-N., Yu J.-G. (2015). Betulin inhibits pro-inflammatory cytokines expression through activation STAT3 signaling pathway in human cardiac cells. Eur. Rev. Med. Pharm. Sci..

[163] Jamila N., Khairuddean M., Yeong K.K., Osman H., Murugaiyah V. (2015). Cholinesterase inhibitory triterpenoids from the bark of Garcinia hombroniana. J. Enzym. Inhib. Med. Chem..

[164] Akihisa T., Ogihara J., Kato J., Yasukawa K., Ukiya M., Yamanouchi S., Oishi K. (2001). Inhibitory effects of triterpenoids and sterols on human immunodeficiency virus-1 reverse transcriptase. Lipids.

[165] Tsai C.W., Tsai R.T., Liu S.P., Chen C.S., Tsai M.C., Chien S.H., Hung H.S., Lin S.Z., Shyu W.C., Fu R.H. (2017). Neuroprotective Effects of Betulin in Pharmacological and Transgenic Caenorhabditis elegans Models of Parkinson’s Disease. Cell Transpl..

[166] Zhou Z., Zhu C., Cai Z., He L., Lou X., Qi X. (2018). Betulin induces cytochrome c release and apoptosis in colon cancer cells via NOXA. Oncol. Lett..

[167] Ullah F., Hussain H., Hussain J., Bukhari I.A., Khan M.T.H., Choudhary M.I., Gilani A.H., Ahmad V.U. (2007). Tyrosinase inhibitory pentacyclic triterpenes and analgesic and spasmolytic activities of methanol extracts of *Rhododendron collettianum*. Phyther. Res..

[168] Goswami P., Paul S., Banerjee R., Kundu R., Mukherjee A. (2018). Betulinic acid induces DNA damage and apoptosis in SiHa cells. Mutat. Res. Toxicol. Env.. Mutagen..

[169] Zhan X.K.A.I., Li J.U.N.L., Zhang S.E.N., Xing P.U.Y., Xia M.F.A.N. (2018). Betulinic acid exerts potent antitumor effects on paclitaxel - resistant human lung carcinoma cells ( H460 ) via G2 / M phase cell cycle arrest and induction of mitochondrial apoptosis. Oncol. Lett..

[170] Gomes Castro A.J., Cazarolli L.H., Bretanha L.C., Sulis P.M., Rey Padilla D.P., Aragón Novoa D.M., Dambrós B.F., Pizzolatti M.G., Mena Barreto Silva F.R. (2018). The potent insulin secretagogue effect of betulinic acid is mediated by potassium and chloride channels. Arch. Biochem. Biophys..

[171] Bossolani G.D.P., Ueda-Nakamura T., Silva S.O., Dias Filho B.P., Costa T.O.G., Quintanilla R.H.R., Martinez S.T., Veiga V.F., Pinto A.C., Nakamura C.V. (2017). Anti-trypanosoma activity and synergistic effects of natural and semi-synthetic triterpenes and predominant cell death through autophagy in amastigote forms. J. Braz. Chem. Soc..

[172] De Almeida P.D.O., Boleti A.P.d.A., Rüdiger A.L., Lourenço G.A., da Veiga Junior V.F., Lima E.S. (2015). Anti-Inflammatory Activity of Triterpenes Isolated from *Protium paniculatum* Oil-Resins. Evid. Based Complement. Altern. Med..

[173] Akihisa T., Franzblau S.G., Ukiya M., Okuda H., Zhang F., Yasukawa K., Suzuki T., Kimura Y. (2005). Antitubercular Activity of Triterpenoids from Asteraceae Flowers. Biol. Pharm. Bull..

[174] Liu K., Qin Y.H., Yu J.Y., Ma H., Song X.L. (2016). 3-rythrodiol isolated from Conyza canadensis inhibits MKN-45 human gastric cancer cell proliferation by inducing apoptosis, cell cycle arrest, DNA fragmentation, ROS generation and reduces tumor weight and volume in mouse xenograft model. Oncol. Rep..

[175] Manayi A., Hadjiakhoondi A., Ardekani M.R.S., Vazirian M., Khanavi M., Saeidnia S., Ostad S.N., Akhtar Y. (2013). Chemical Constituents and Cytotoxic Effect of the Main Compounds of *Lythrum salicaria* L.. Z. Fur Nat. Sect. C J. Biosci..

[176] Martín R., Miana M., Jurado-López R., Martínez-Martínez E., Gómez-Hurtado N., Delgado C., Bartolomé M., Román J.A., Cordova C., Lahera V. (2012). Diol triterpenes block profibrotic effects of angiotensin II and protect from cardiac hypertrophy. PLoS ONE.

[177] Martín R., Hernández M., Córdova C., Nieto M.L. (2012). Natural triterpenes modulate immune-inflammatory markers of experimental autoimmune encephalomyelitis: Therapeutic implications for multiple sclerosis. Br. J. Pharm..

[178] Wansi J.D., Chiozem D.D., Tcho A.T., Toze F.A.A., Devkota K.P., Ndjakou B.L., Wandji J., Sewald N. (2010). Antimicrobial and antioxidant effects of phenolic constituents from *Klainedoxa gabonensis*. Pharm. Biol..

[179] Máñez S., Recio M.C., Giner R.M., Ríos J.L. (1997). Effect of selected triterpenoids on chronic dermal inflammation. Eur. J. Pharm..

[180] Kinoshita K., Yang Y., Koyama K., Takahashi K., Nishino H. (1999). Inhibitory effect of some triterpenes from cacti on 32Pi-incorporation into phospholipids of HeLa cells promoted by 12-0-tetradecanoylphorbol-13-acetate. Phytomedicine.

[181] Yasukawa K., Akihisa T., Oinuma H., Kasahara Y., Kimura Y., Yamanouchi S., Kumaki K., Tamura T., Takido M. (1996). Inhibitory effect of di- and trihydroxy triterpenes from the flowers of compositae on 12-O-tetradecanoylphorbol-13-acetate-induced inflammation in mice. Biol. Pharm. Bull..

[182] Aragão G.F., Carneiro L.M.V., Junior A.P.F., Vieira L.C., Bandeira P.N., Lemos T.L.G., Viana G.S.d.B. (2006). A possible mechanism for anxiolytic and antidepressant effects of alpha- and beta-amyrin from Protium heptaphyllum (Aubl.) March. Pharm. Biochem. Behav..

[183] Xu F., Huang X., Wu H., Wang X. (2018). Beneficial health effects of lupenone triterpene: A review. Biomed. Pharm..

[184] Çevik D., Burçin Yılmazgöz Ş., Kan Y., Akhan Güzelcan E., Durmaz I., Çetin-Atalay R., Kırmızıbekmez H. (2018). Bioactivity-guided isolation of cytotoxic secondary metabolites from the roots of Glycyrrhiza glabra and elucidation of their mechanisms of action. Ind. Crop. Prod..

[185] Subba Rao G., Sinsheimer J.E., Cochran K.W. (1974). Antiviral activity of triterpenoid saponins containing acylated β-amyrin aglycones. J. Pharm. Sci..

[186] Wei C.C., Chang C.H., Liao V.H.C. (2017). Anti-Parkinsonian effects of β-amyrin are regulated via LGG-1 involved autophagy pathway in Caenorhabditis elegans. Phytomedicine.

[187] Zhang Y., Jayaprakasam B., Seeram N.P., Olson L.K., DeWitt D., Nair M.G. (2004). Insulin Secretion and Cyclooxygenase Enzyme Inhibition by Cabernet Sauvignon Grape Skin Compounds. J. Agric. Food Chem..

[188] Rivero-Cruz J.F., Zhu M., Kinghorn A.D., Wu C.D. (2008). Antimicrobial constituents of Thompson seedless raisins (Vitis vinifera) against selected oral pathogens. Phytochem. Lett..

[189] Thao N.P., Luyen B.T.T., Koo J.E., Kim S., Koh Y.S., Van Thanh N., Cuong N.X., Van Kiem P., Van Minh C., Kim Y.H. (2016). In vitro anti-inflammatory components isolated from the carnivorous plant Nepenthes mirabilis (Lour.) Rafarin. Pharm. Biol..

[190] Ramírez-Espinosa J.J., García-Jiménez S., Rios M.Y., Medina-Franco J.L., López-Vallejo F., Webster S.P., Binnie M., Ibarra-Barajas M., Ortiz-Andrade R., Estrada-Soto S. (2013). Antihyperglycemic and sub-chronic antidiabetic actions of morolic and moronic acids, in vitro and in silico inhibition of 11β-HSD 1. Phytomedicine.

[191] Giner-Larza E.M., Máñez S., Giner R.M., Recio M.C., Prieto J.M., Cerdá-Nicolás M., Ríos J.L. (2002). Anti-inflammatory triterpenes from Pistacia terebinthus galls. Planta Med..

[192] Gurovic M.S.V., Castro M.J., Richmond V., Faraoni M.B., Maier M.S., Murray A.P. (2010). Triterpenoids with acetylcholinesterase inhibition from chuquiraga erinacea D. Don. subsp. erinacea (Asteraceae). Planta Med..

[193] Castro M.J., Richmond V., Romero C., Maier M.S., Estévez-Braun A., Ravelo Á.G., Faraoni M.B., Murray A.P. (2014). Preparation, anticholinesterase activity and molecular docking of new lupane derivatives. Bioorganic Med. Chem..

[194] Okahara F., Suzuki J., Hashizume K., Osaki N., Shimotoyodome A. (2016). Triterpene alcohols and sterols from rice bran reduce postprandial hyperglycemia in rodents and humans. Mol. Nutr. Food Res..

[195] Radha M., Laxmipriya N. (2015). Evaluation of biological properties and clinical effectiveness of Aloe vera: A systematic review. J. Tradit. Complement. Med..

[196] Ikeda I., Nakashima-Yoshida K., Sugano M. (1985). Effects of Cycloartenol on Absorption and Serum Levels of Cholesterol in Rats. J. Nutr. Sci. Vitam..

[197] Momo I., Kuete V., Dufat H., Michel S., Wandji J. (2011). Antimicrobial Activity of the Methanolic Extract and Compounds From the Stem Bark of Garcinia Lucida Vesque (Clusiaceae). Int. J. Pharm. Pharm. Sci..

[198] Benmerache A., Alabdul Magid A., Labed A., Kabouche A., Voutquenne-Nazabadioko L., Hubert J., Morjani H., Kabouche Z. (2017). Isolation and characterisation of cytotoxic compounds from Euphorbia clementei Boiss. Nat. Prod. Res..

[199] Pujirahayu N., Bhattacharjya D.K., Suzuki T., Katayama T. (2019). α-Glucosidase Inhibitory Activity of Cycloartane-Type Triterpenes Isolated from Indonesian Stingless Bee Propolis and Their Structure–Activity Relationship. Pharmaceuticals.

[200] Niu H., Li X., Yang A., Jin Z., Wang X., Wang Q., Yu C., Wei Z., Dou C. (2018). Cycloartenol exerts anti-proliferative effects on Glioma U87 cells via induction of cell cycle arrest and p38 MAPK-mediated apoptosis. JBUON.

[201] Tung N.H., Kwon H.J., Kim J.H., Ra J.C., Kim J.A., Kim Y.H. (2010). An anti-influenza component of the bark of alnus japonica. Arch. Pharm. Res..

[202] (2019). ACD/ChemSketch.

[203] Thomsen R., Christensen M.H. (2006). MolDock: A New Technique for High-Accuracy Molecular Docking. J. Med. Chem..

[204] Gupta K., Selinsky B.S., Kaub C.J., Katz A.K., Loll P.J. (2004). The 2.0 A resolution crystal structure of prostaglandin H2 synthase-1: Structural insights into an unusual peroxidase. J. Mol. Biol..

[205] Duggan K.C., Walters M.J., Musee J., Harp J.M., Kiefer J.R., Oates J.A., Marnett L.J. (2010). Molecular basis for cyclooxygenase inhibition by the non-steroidal anti-inflammatory drug naproxen. J. Biol. Chem..

[206] Oyama T., Toyota K., Waku T., Hirakawa Y., Nagasawa N., Kasuga J., Hashimoto Y., Miyachi H., Morikawa K. (2009). IUCr Adaptability and selectivity of human peroxisome proliferator-activated receptor (PPAR) pan agonists revealed from crystal structures. Acta Cryst. Sect. D Biol. Cryst..

[207] Andersen H.S., Iversen L.F., Jeppesen C.B., Branner S., Norris K., Rasmussen H.B., Møller K.B., Møller N.P. (2000). 2-(oxalylamino)-benzoic acid is a general, competitive inhibitor of protein-tyrosine phosphatases. J. Biol. Chem..

[208] Fradera X., Vu D., Nimz O., Skene R., Hosfield D., Wynands R., Cooke A.J., Haunso A., King A., Bennett D.J. (2010). X-ray structures of the LXRalpha LBD in its homodimeric form and implications for heterodimer signaling. J. Mol. Biol..

[209] Williams S., Bledsoe R.K., Collins J.L., Boggs S., Lambert M.H., Miller A.B., Moore J., McKee D.D., Moore L., Nichols J. (2003). X-ray crystal structure of the liver X receptor beta ligand binding domain: Regulation by a histidine-tryptophan switch. J. Biol. Chem..

[210] Cheung J., Rudolph M.J., Burshteyn F., Cassidy M.S., Gary E.N., Love J., Franklin M.C., Height J.J. (2012). Structures of human acetylcholinesterase in complex with pharmacologically important ligands. J. Med. Chem..

[211] Berman H.M., Westbrook J., Feng Z., Gilliland G., Bhat T.N., Weissig H., Shindyalov I.N., Bourne P.E. (2000). The Protein Data Bank. Nucleic Acids Res..

[212] Ramírez-Espinosa J.J., Rios M.Y., Paoli P., Flores-Morales V., Camici G., Rosa-Lugo V.d.l., Hidalgo-Figueroa S., Navarrete-Vázquez G., Estrada-Soto S. (2014). Synthesis of oleanolic acid derivatives: In vitro, in vivo and in silico studies for PTP-1B inhibition. Eur. J. Med. Chem..

[213] Tarr G.S., Reuter H. (2015). Review of the safety of nonsteroidal anti-inflammatory drugs and selective cyclo-oxygenase-2 inhibitors. South. Afr. Fam. Pr..

[214] Hitender S., Pushpander K., Deshmukh R., Anupam B., Sunil K. (2018). Pentacyclic triterpenes: New tools to fight metabolic syndrome. Phytomedicine.

[215] Yang F., Liang-Wei Z., Mei-Tang F., Ai-Hong L., Jia L., Tian-Sheng Z., Xiao-Ping L., Bin W., Yue-Wei G., Shui-Chun M. (2018). Dictyoptesterols A–C, ∆22-24-oxo cholestane-type sterols with potent PTP1B inhibitory activity from the brown alga Dictyopteris undulata Holmes. Fitoterapia.

[216] Na B., Nguyen P.-H., Zhao B.-T., Vo Q.-H., Min B.S., Woo M.H. (2016). Protein tyrosine phosphatase 1B (PTP1B) inhibitory activity and glucosidase inhibitory activity of compounds isolated from *Agrimonia pilosa*. Pharm. Biol..

[217] Moustafa E.M., Thabet N.M. (2017). Beta-sitosterol upregulated paraoxonase-1 via peroxisome proliferator-activated receptor-γ in irradiated rats. Can. J. Physiol. Pharm..

[218] Puius Y.A., Zhao Y., Sullivan M., Lawrence D.S., Almo S.C., Zhang Z.Y. (1997). Identification of a second aryl phosphate-binding site in protein-tyrosine phosphatase 1B: A paradigm for inhibitor design. Proc. Natl. Acad. Sci. USA.

[219] Shah M.R., Ishtiaq, Hizbullah S.M., Habtemariam S., Zarrelli A., Muhammad A., Collina S., Khan I. (2016). Protein tyrosine phosphatase 1B inhibitors isolated from *Artemisia roxburghiana*. J. Enzym. Inhib. Med. Chem..

[220] Rigano D., Sirignano C., Taglialatela-Scafati O. (2017). The potential of natural products for targeting PPARα. Acta Pharm. Sin. B.

[221] Hiebl V., Ladurner A., Latkolik S., Dirsch V.M. (2018). Natural products as modulators of the nuclear receptors and metabolic sensors LXR, FXR and RXR. Biotechnol. Adv..

