# Supplementary material for: Chemistry, Biological Activities and In Silico Bioprospection of Sterols and Triterpenes from Mexican Columnar Cactaceae
Source: Molecules. 2020 Apr 3;25(7):1649. doi: 10.3390/molecules25071649 (PMC7180492; doi:10.3390/molecules25071649)
Supplement: Supplementary file 1 [file molecules-25-01649-s001.pdf]

molecules

MDPI

Review

Chemistry, biological activities and *in silico* bioprospection of sterols and triterpenes from Mexican columnar Cactaceae

Juan Rodrigo Salazar[1]*; Marco A. Loza-Mejía[1]* and Diego Soto-Cabrera[1,2]

[1] Design, Isolation, and Synthesis of Bioactive Molecules Research Group, Chemical Sciences Faculty, Universidad La Salle. Benjamín Franklin 45, 06140, Mexico City, Mexico; J.R.S. juan.salazar@lasalle.mx; M.A.L.-M. marcoantonio.loza@lasalle.mx

[2] Affiliation 2; e-mail@e-mail.com

* Correspondence: J.R.S. juan.salazar@lasalle.mx; M.A.L.-M. marcoantonio.loza@lasalle.mx; Tel.: +52-55-5278-9500 (J.R.S. & M.A.L.-M.)

Received: date; Accepted: date; Published: date

Abstract: The Cactaceae family is an important source of triterpenes and sterols. The widely uses of those plants include food, gathering, medicinal, and live fences. Several studies had led to the isolation and characterization of many bioactive compounds. This review is focused on the chemistry and biological properties of sterols and triterpenes isolated mainly from columnar species of Mexican Cactaceae. Regard the biological properties of those compounds, until a few cases, their molecular mechanisms displayed are not still fully understand. To contribute to the above, computational chemistry tools have given a boost to traditional methods used in natural products research, allowing a more comprehensive exploration of chemistry and biological activities of isolated compounds and extracts. In this work, we discuss the chemical diversity of sterols and triterpenes isolated from Cactaceae and their biological activities. From this information an *in silico* bioprospection was carried out; the results suggest that sterols and triterpenoids present in Cactaceae have interesting substitution patterns that allow them to interact with some bio targets related to inflammation, metabolic diseases, and neurodegenerative processes; thus they should be considered as attractive leads for the development of drugs for the management of chronic degenerative diseases.

Keywords: Cactaceae; Bioprospection; Sterol; Triterpene; Bioactivity; in silico screening; Inflammation; Diabetes.

Supplementary Materials: The following are available online at www.mdpi.com/xxx/s1, Table S1: Complete docking results from the in silico bioprospection on compounds isolated from Mexican Columnar Cactaceae.

Table S1. Complete docking results from the in silico bioprospection on compounds isolated from Mexican Columnar Cactaceae.

Ligand	COX-1	COX-2	PTP-1B	PPAR-α	PPAR-γ	LXR-α	LXR-β	AChE
Thurberol	-132.1	-144.1	-135.3	-129.3	-140.4	-161.9	-158.7	-147.5
Locereol	-133.1	-141.1	-122.2	-135.3	-137.7	-152.5	-155.8	-146.3
Fucosterol	-130.5	-141.7	-141.7	-135.7	-138.9	-167.1	-167.7	-152.9
5α-Cholesta-8,14-dien-3β-ol	-130.4	-141.5	-124.0	-132.1	-136.0	-152.8	-154.8	-140.5
Spinasterol	-130.6	-138.5	-133.1	-140.8	-131.2	-157.0	-158.2	-152.6
24-Methylencholesterol	-127.4	-139.9	-132.7	-133.5	-136.0	-163.1	-157.8	-148.6

β-Sitosterol	-124.3	-136.8	-131.6	-140.9	-142.4	-165.2	-164.2	-151.7
Peniocerol	-124.3	-134.1	-132.4	-127.9	-140.5	-158.6	-155.8	-143.4
24-Methylenelofenol	-127.3	-130.7	-124.1	-131.9	-126.4	-155.7	-154.6	-146.2
Lophenol	-123.7	-131.2	-119.4	-130.9	-129.7	-143.1	-146.0	-142.0
Deoxyviperidone	-120.7	-132.9	-121.9	-127.3	-131.5	-151.1	-152.2	-141.3
Lathosterol	-122.0	-131.2	-124.1	-124.1	-129.4	-147.9	-146.0	-141.2
Scottenol	-121.2	-131.7	-132.9	-139.0	-138.9	-155.9	-153.6	-147.4
5α-Campest-7-en-3-ol	-119.8	-129.4	-122.4	-128.6	-128.6	-151.3	-146.7	-143.2
Cyclostenol	-117.2	-129.4	-140.4	-137.2	-126.4	-155.4	-158.2	-147.7
5β-Deoxyviperidone	-118.7	-125.5	-126.9	-128.2	-127.4	-144.3	-150.5	-148.4
Opuntisterol	-119.8	-124.3	-126.0	-141.5	-132.7	-155.9	-157.9	-152.1
24-Dehydropollinasterol	-114.3	-125.9	-130.2	-132.4	-123.2	-150.6	-152.3	-139.6
Steneocerol	-111.4	-123.9	-125.7	-123.3	-122.8	-144.8	-144.1	-141.5
Steneocerol	-113.4	-119.4	-133.0	-136.2	-126.2	-150.3	-146.5	-147.4
Macdougallin	-109.1	-121.3	-126.3	-125.9	-120.8	-145.0	-144.8	-135.1
25,27-Dehydrolanosterol	-106.8	-120.2	-122.1	-126.9	-119.8	-145.5	-144.1	-141.1
Viperidone	-110.5	-114.8	-110.3	-119.8	-130.7	-148.2	-147.6	-141.4
Cycloartenol	-108.3	-115.6	-118.8	-125.9	-122.0	-155.3	-154.9	-143.9
Viperidinone	-103.7	-109.3	-104.1	-119.9	-118.9	-144.6	-144.0	-129.8
Lupenone	-104.0	-97.1	-98.4	-119.8	-111.6	-145.7	-147.0	-134.2
Thurberogenin	-92.3	-93.6	-104.8	-123.4	-114.8	-153.9	-155.5	-143.2
Lupeol	-97.7	-87.3	-97.7	-114.2	-108.2	-146.7	-148.7	-129.8
Betulinic aldehyde	-94.9	-88.6	-97.8	-120.2	-107.0	-141.6	-150.6	-119.3
16-Hydroxybetulinic acid	-82.9	-95.1	-98.8	-117.0	-109.3	-145.4	-156.1	-127.6
Calenduladiol	-92.8	-85.1	-93.4	-120.1	-108.8	-147.8	-152.9	-127.8
16-Hydroxystellatogenin	-93.8	-83.0	-98.5	-128.7	-119.5	-149.2	-152.3	-134.8
22-Hydroxystellatogenin	-94.0	-79.3	-93.4	-129.9	-112.6	-138.1	-145.1	-135.5
21-Ketobetulinic acid	-81.6	-87.0	-97.6	-112.4	-108.4	-131.0	-156.2	-124.8
Machaerogenin	-87.0	-79.4	-90.8	-111.9	-101.3	-134.4	-138.5	-117.5
Stellatogenin	-83.1	-80.8	-99.4	-124.8	-116.9	-142.9	-150.5	-139.3
β-Amyrin	-88.3	-74.3	-98.6	-111.1	-112.5	-137.3	-138.5	-121.8
Erythrodiol	-87.4	-74.4	-97.4	-112.7	-108.4	-139.4	-133.3	-117.2
Betulin	-85.6	-74.8	-99.1	-116.2	-109.2	-141.4	-153.0	-133.0
Betulinic acid	-81.2	-78.2	-100.1	-124.3	-106.4	-141.3	-155.2	-137.3
Oleanolic aldehyde	-87.9	-71.3	-100.4	-116.8	-115.6	-139.1	-145.2	-129.0
Longispinogenin	-88.9	-69.7	-87.2	-107.6	-98.7	-131.0	-136.1	-117.3
Gummosogenin	-79.9	-78.5	-96.0	-114.1	-106.2	-142.1	-139.0	-126.0
Alamosogenin	-81.0	-76.9	-98.1	-120.5	-118.2	-141.6	-144.0	-138.3
Morolic acid	-81.2	-72.9	-93.0	-110.9	-108.2	-142.6	-138.6	-120.4
Cochalic acid	-73.2	-80.1	-88.0	-101.4	-96.7	-118.7	-137.1	-130.5
Oleanolic acid	-80.9	-71.0	-101.6	-118.1	-114.2	-127.8	-141.3	-133.2

Treleasegenic acid	-80.9	-69.2	-99.7	-115.5	-111.1	-135.8	-143.7	-120.8
Queretaroic acid	-80.3	-68.9	-99.0	-113.0	-114.1	-130.8	-148.8	-136.1
Macheric acid	-82.0	-65.2	-105.2	-112.3	-115.6	-135.3	-138.2	-129.1
Machaeric acid	-81.6	-64.8	-103.8	-114.0	-114.8	-132.9	-140.4	-128.1
Chichipegenin	-77.7	-67.9	-93.2	-121.0	-113.4	-140.2	-138.3	-131.1
Machaerinic acid	-80.5	-64.7	-98.9	-118.0	-109.9	-130.7	-134.4	-118.6
Dumortierigenin	-80.4	-64.3	-87.6	-105.4	-102.8	-133.7	-143.1	-123.7
Olean-12-ene-3β,16β,22α-triol	-75.3	-69.4	-91.7	-96.2	-94.6	-123.9	-129.1	-100.6
Mirtillogenic acid	-76.3	-64.2	-95.4	-123.7	-119.3	-134.2	-145.1	-130.8
Desoxyfillirigenin	-77.4	-60.1	-80.7	-109.3	-92.9	-140.6	-130.6	-110.9
3β-Hydroxy-11α,12α-epoxyolean-2,8,13β-olide	-72.5	-62.2	-89.6	-104.5	-103.9	-117.7	-140.9	-126.7
Maniladiol	-70.9	-61.5	-87.9	-101.4	-98.9	-126.6	-126.7	-110.0
Pachanol	-65.9	-61.7	-74.2	-99.0	-91.8	-135.4	-135.3	-100.1

© 2020 by the authors. Submitted for possible open access publication under the terms and conditions of the Creative Commons Attribution (CC BY) license (http://creativecommons.org/licenses/by/4.0/).
